# Glycine and Glucose Exert Divergent Concentration-Dependent Interfacial Regulatory Effects on Goethite-Catalyzed Polyphenol–Maillard Abiotic Humification

**DOI:** 10.3390/molecules31183302

**Published:** 2026-09-17

**Authors:** Nan Wang, Zihan Zheng, Mingshuo Wang, Jiawen Peng, Houfu Chen, Shuai Wang

**Affiliations:** 1 College of Agriculture, Jilin Agricultural Science and Technology College, Jilin 132101, China; wangnan664806@126.com (N.W.); 13341501075@163.com (Z.Z.); wangmingshuo@jlnku.edu.cn (M.W.); pjw22_jlastu@163.com (J.P.); 2 College of Agriculture, Jilin Agricultural University, Changchun 130118, China; 3 Department of Chemical Engineering, University of Waterloo, Waterloo, ON N2L 3G1, Canada; h534chen@uwaterloo.ca

**Keywords:** abiotic humification, Maillard reaction, goethite, glycine, glucose

## Abstract

Iron oxide-mediated abiotic humification is critical for long-term soil organic carbon (SOC) stabilization. Goethite (α-FeOOH), the most thermodynamically stable iron oxyhydroxide in terrestrial ecosystems, is proposed to catalyze coupled Maillard reaction and polyphenol oxidation via surface hydroxyl and Fe(III) active sites. Nevertheless, the divergent interfacial regulatory mechanisms of glycine (Gly, N source) and glucose (Glu, C source) concentration gradients in goethite-catalyzed polyphenol-Maillard systems remain poorly understood. Two independent 360-h gradient incubation experiments were performed using synthetic goethite as a catalyst and catechol as a model polyphenol precursor to probe their concentration-dependent effects on short-term interfacial transformation kinetics and humic-like product properties. Kinetics, product properties, and underlying pathways were characterized via Gaussian and first-order asymptotic modeling, humic-like acid (HLA)/fulvic-like acid (FLA) fractionation, elemental analysis, Fourier-transform infrared (FTIR) spectroscopy, and partial least squares structural equation modeling (PLS-SEM). Results showed that 0.12 mol/L was the optimal concentration for both precursors to accelerate intermediate transformation. Gly elevated the baseline aromaticity parameter y_0_ by 108.87% (vs. 20.38% for Glu), while Glu increased the asymptotic maximum dissolved organic carbon (DOC) by 322.66% (vs. 78.02% for Gly). Moderate Gly (0.03 mol/L) yielded the highest C_HLA_. Excessive precursors reduced the C_HLA_/C_FLA_ ratio via interfacial competitive adsorption and site occupation. The two precursors exerted opposing effects on the hydroxyl stretching peak of humic products, but both induced goethite surface reconstruction and activated iron-bearing surface functional groups. PLS-SEM confirmed precursor concentration as the dominant driver of humification kinetics. This study clarifies the distinct roles of Gly in aromatic cyclization and Glu as an aliphatic C donor during short-term mineral–organic interfacial processes, providing quantitative benchmarks for optimizing artificial humus production.

## 1. Introduction

Abiotic humification is a critical pathway for the long-term sequestration of soil organic carbon (SOC) [1,2]. It converts labile low-molecular-weight precursors (including reducing sugars, amino acids, and polyphenols) into recalcitrant humic-like substances (HLS) via non-enzymatic polymerization, and plays an irreplaceable role in mitigating climate change and improving soil fertility [3,4]. Among various abiotic humification mediators, iron oxides have emerged as core regulators of SOC turnover due to their widespread distribution, strong adsorption capacity, and oxidative catalytic activity [3,5,6]. Goethite (α-FeOOH), as the most thermodynamically stable and abundant iron oxyhydroxide in terrestrial ecosystems, exhibits a unique dual adsorption–oxidation catalytic effect [7]. Its abundant surface hydroxyl groups and Fe(III) active sites can effectively enrich organic precursors and are proposed to accelerate interfacial electron transfer, significantly promoting the Maillard reaction and polyphenol oxidative polymerization [7]. The integrated polyphenol–Maillard pathway is the abiotic humification route most representative of natural soil conditions, and its reaction kinetics and product properties are strongly modulated by precursor composition, including the C/N ratio and precursor type [5]. Therefore, systematically investigating the effects of different precursor concentrations on goethite-mediated integrated polyphenol–Maillard reactions is of great theoretical significance and practical value for elucidating SOC sequestration mechanisms and optimizing artificial humus (HS) production processes.

Recent research on mineral-mediated abiotic humification has progressed from macroscopic yield quantification to molecular-level mechanistic dissection, and the catalytic function of iron oxides has become a focal research topic [8,9]. Accumulating evidence indicates that hydroxyl groups on iron oxide surfaces concentrate precursors such as glucose (Glu), glycine (Gly), and polyphenols via hydrogen bonding or ligand exchange, while surface Fe(III) sites function as electron acceptors to mediate oxidative polymerization and accelerate HLS generation [10,11]. Notably, sorption is not merely a preliminary step of the reaction, but a core regulatory process: precursors differ in molecular charge, functional group type and binding affinity to heterogeneous iron oxyhydroxide surfaces, which directly modulates local surface concentration, competitive site occupation, product retention and the measured distribution of dissolved, HLA and FLA fractions [11]. For instance, oxygen vacancy defects on hematite surfaces enhance Lewis acid site activity, reduce the activation energy barrier of the Maillard reaction, and promote the formation of aromatic geopolymers [12]; ferrihydrite triggers non-enzymatic browning reactions via Fe(III)–polyphenol complexation, significantly elevating humification efficiency [5]. Regarding reaction pathways, the Maillard reaction and polyphenol oxidative polymerization are recognized as two core abiotic humification routes, and they exert pronounced synergistic effects in natural soil environments [8]. Quinones, the primary oxidation products of polyphenols, can cross-link with Maillard reaction intermediates to form HLS with more complex molecular structures and greater chemical stability [12]. Precursor composition, as a key factor regulating humification, has received extensive attention for its effects on reaction kinetics and product properties. Gly, a typical amino acid precursor, underwent nucleophilic addition reactions with carbonyl groups of Glu through its amino groups to generate N-containing aromatic heterocycles, significantly enhancing the aromaticity and stability of HLS [13]. By contrast, Glu, as the primary carbon source precursor, provides the aliphatic carbon skeleton of HLS through ring-opening, dehydration, and polymerization reactions, and determines the overall carbon sequestration capacity of the reaction system [14]. Furthermore, previous studies have demonstrated that precursor concentration and C/N ratio modulate humification by regulating adsorption competition at the mineral interface and the coupling efficiency between reaction pathways [15]. Nevertheless, existing studies have predominantly focused on the catalytic effects of single precursors or reaction processes under a fixed C/N ratio. Systematic comparative investigations into the divergent regulatory effects of Gly and Glu concentration gradients in the integrated polyphenol–Maillard system, as well as their impacts on the interfacial reaction mechanisms of goethite, remain scarce.

Despite recent progress in mineral-mediated abiotic humification research, most studies have centered on the structural properties of iron oxides rather than systematic concentration-dependent regulation by organic precursors. Accordingly, in the integrated polyphenol–Maillard system—the pathway most representative of natural soil conditions—the mechanisms by which Gly and Glu concentration gradients modulate reaction kinetics and HLS properties via goethite interfacial interactions remain poorly understood. Few studies have disentangled their distinct regulatory roles beyond single-precursor or fixed C/N ratio systems. Building on these knowledge gaps, this study employed goethite as the model catalyst and catechol (Cat) as the model polyphenol precursor. Two independent gradient incubation experiments were designed: (1) a Gly gradient series with varying Gly concentrations (0, 0.03, 0.06, 0.12, and 0.24 mol/L) and fixed Glu and Cat concentrations (0.06 mol/L each); (2) a Glu gradient series with varying Glu concentrations (0, 0.03, 0.06, 0.12, and 0.24 mol/L) and fixed Gly and Cat concentrations (0.06 mol/L each). Using kinetic modeling, elemental analysis, Fourier-transform infrared (FTIR) spectroscopy, and Partial least squares structural equation modeling (PLS-SEM), we systematically investigated the effects of Gly and Glu concentration gradients on short-term goethite-mediated interfacial transformation and polyphenol-Maillard coupled reaction characteristics, clarified compositional and structural differences in HLS products, and revealed the underlying interfacial catalytic mechanisms. The primary aim of this study was to quantitatively disentangle the divergent concentration-dependent interfacial regulatory mechanisms of nitrogenous Gly and Glu precursors in goethite-catalyzed coupled polyphenol–Maillard abiotic humification systems. The key novelties of this work are threefold: (1) it systematically compares the independent concentration gradients of Gly and Glu under a unified polyphenol-Maillard framework, rather than relying on fixed C/N ratios or single-precursor experimental designs; (2) it combines Gaussian and asymptotic kinetic modeling with PLS-SEM to decouple the kinetic, compositional, and structural pathways driving carbon sequestration; and (3) it directly links precursor-induced goethite surface reconstruction to humification performance, providing mechanistic evidence for active mineral–organic co-evolution during abiotic humification. This study provides mechanistic references for elucidating SOC sequestration mechanisms and optimizing artificial HS production processes, and the conclusions are limited to short-term reaction systems.

## 2. Results

### 2.1. Supernatant E_4_/E_6_ Ratio and Dissolved Organic C (DOC) Dynamics

To quantitatively characterize the kinetic features of goethite-mediated abiotic humification and extract characteristic parameters reflecting reaction rate and intermediate transformation, the temporal dynamics of the supernatant E_4_/E_6_ ratio were fitted using a baseline-corrected unimodal Gaussian model (Equation (1)). This model is widely used to describe the unimodal dynamic pattern of intermediate accumulation and subsequent polymerization during humification. Its mathematical expression is as follows:

(1) $$y\;=\;y_{0}\;+\;\frac{A}{w\sqrt{\frac{\pi }{4\ln2}}}\exp\left(-\frac{4\ln2\cdot {\left(x\;-\;x_{c}\right)}^{2}}{w^{2}}\right)$$

where *y* is the dependent variable representing the E_4_/E_6_ ratio of the supernatant at reaction time x; *y*_0_ is the baseline constant of the E_4_/E_6_ ratio, reflecting the intrinsic aromatic condensation degree of newly formed HLS; *x* is the independent variable corresponding to reaction time (h); *x_c_* is the center position of the Gaussian peak, denoting the time at which low-aromatic, low-molecular-weight intermediates reach maximum accumulation; *A* is the peak amplitude parameter, positively correlated with the total increment in E_4_/E_6_ signal intensity during the reaction; *w* is the full width at half maximum (FWHM) of the Gaussian peak, characterizing the duration of the humification active phase. A smaller *w* value indicates faster intermediate transformation and higher overall reaction efficiency.

Among the model parameters, *y*_0_ is associated with the aromaticity of nascent HLS, *x_c_* represents the peak accumulation time of small-molecule and low-aromatic intermediate products, and *w* characterizes the duration of the “active period” in humification—with a narrower peak indicating faster accumulation and transformation of intermediates, thus a shorter active period and higher reaction efficiency.

**Figure 1:**
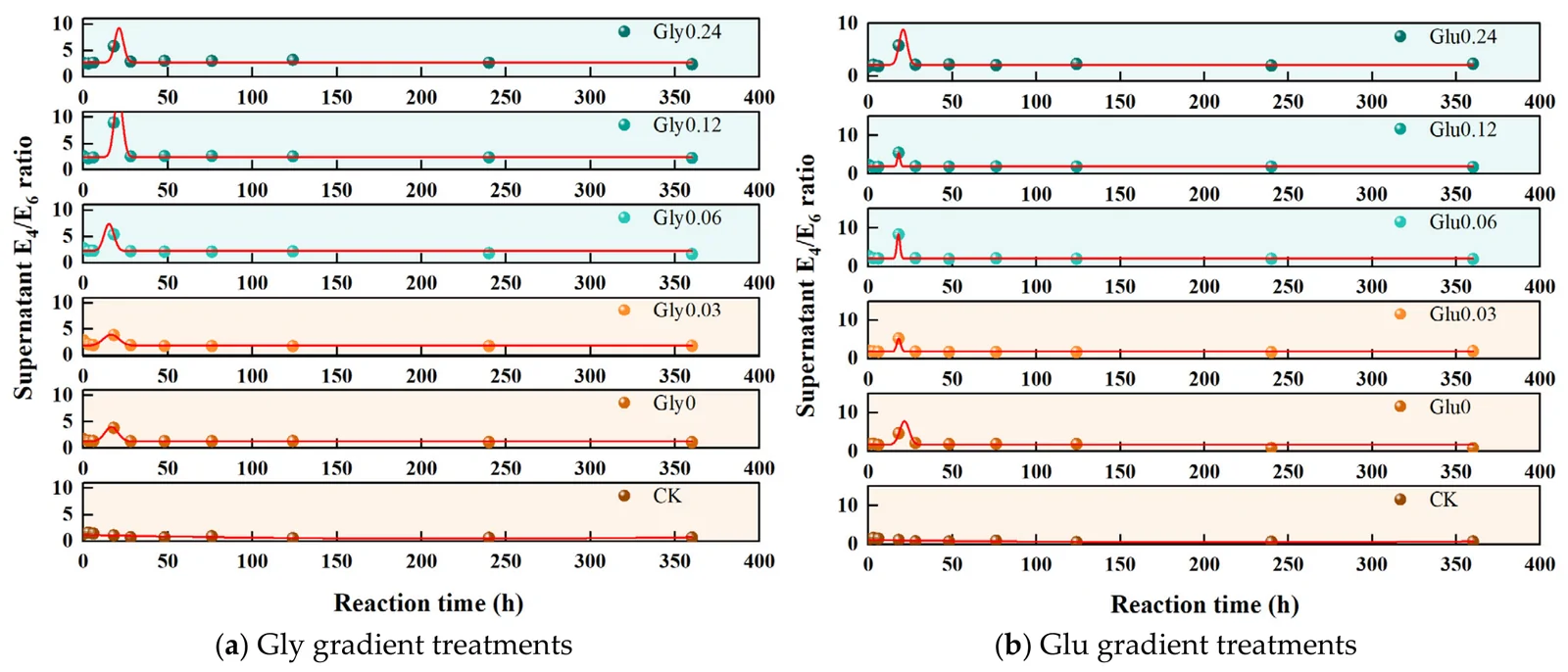
Temporal dynamics of the supernatant E_4_/E_6_ ratio under different Gly (**a**) and Glu (**b**) concentration gradients.

As shown in Figure 1, the Gaussian model provided good fits for the E_4_/E_6_ ratio dynamics during humification across all treatments. The E_4_/E_6_ ratio is a widely used indicator of the aromatic condensation degree of HLS: a higher ratio corresponds to a lower degree of aromatic condensation and a relatively simpler molecular structure of HLS [16]. As presented in Table 1, *y*_0_ exhibited a significant monotonic increasing trend with rising Gly concentration, ranging from 1.31039 to 2.73717, with a relative increase of 108.87%. In contrast, *y*_0_ increased only slightly with rising Glu concentration, varying from 1.75788 to 2.11625, with a relative increase of 20.38%, which was markedly lower than that in the Gly series. For parameter *x_c_*, both the Gly and Glu series exhibited a similar unimodal trend, decreasing first and then increasing with rising precursor concentration [13,16]. In the Gly series, *x_c_* decreased from 16.61733 h to a minimum of 15.15471 h at the medium concentration, and then rebounded to 21.09759 h at the highest concentration. Similarly, in the Glu series, *x_c_* decreased from 21.43066 h to a minimum of 17.81478 h at the medium concentration, and subsequently increased to 20.62358 h at the highest concentration. For parameter *w*, the 0.12 mol/L treatment yielded the smallest value in both the Gly and Glu series, representing the highest reaction efficiency. When precursor concentration was either lower or higher than 0.12 mol/L, *w* values increased significantly, indicating a prolonged intermediate transformation period. Notably, the Gaussian model exhibited poor fitting performance for the CK group (R^2^ = 0.58) with poorly constrained, physically meaningless parameter estimates, as no typical unimodal dynamic of intermediate accumulation and transformation occurred in the absence of organic precursors. Accordingly, the Gaussian parameters of the CK group were not used for quantitative interpretation or cross-treatment comparisons, and were only included to demonstrate the absence of humification activity in the blank system.

To quantify the kinetic characteristics of DOC accumulation and the asymptotic DOC generation potential of the goethite-mediated humification system, the temporal profile of supernatant DOC was fitted using a first-order asymptotic growth model (Equation (2)). This model describes the saturating kinetic pattern of C transformation, where DOC increases rapidly in the early reaction stage and gradually approaches a stable plateau as labile precursors are depleted. Its mathematical expression is as follows:

(2) $$y\;=\;a\;+\;bc^{x}$$

where *y* is the dependent variable representing the DOC concentration in the supernatant at reaction time *x*; *x* is the independent variable corresponding to reaction time (h); *a* is the asymptotic parameter, denoting the theoretical maximum DOC concentration as reaction time approaches infinity, which reflects the potential of the system to accumulate dissolved humic-like products during incubation; *b* is the amplitude parameter, equal to *y*_0_-*a* where *y*_0_ is the initial DOC concentration at *x* = 0. Since initial DOC is lower than the asymptotic maximum, *b* is consistently negative; its absolute value represents the DOC increment from the initial state to the final asymptotic plateau, characterizing the overall magnitude of soluble precursor conversion to dissolved humic-like products; *c* is a rate-related constant (0 < *c* < 1). A smaller *c* value indicates a faster approach to the asymptotic plateau, corresponding to higher kinetic efficiency of C sequestration.

**Figure 2:**
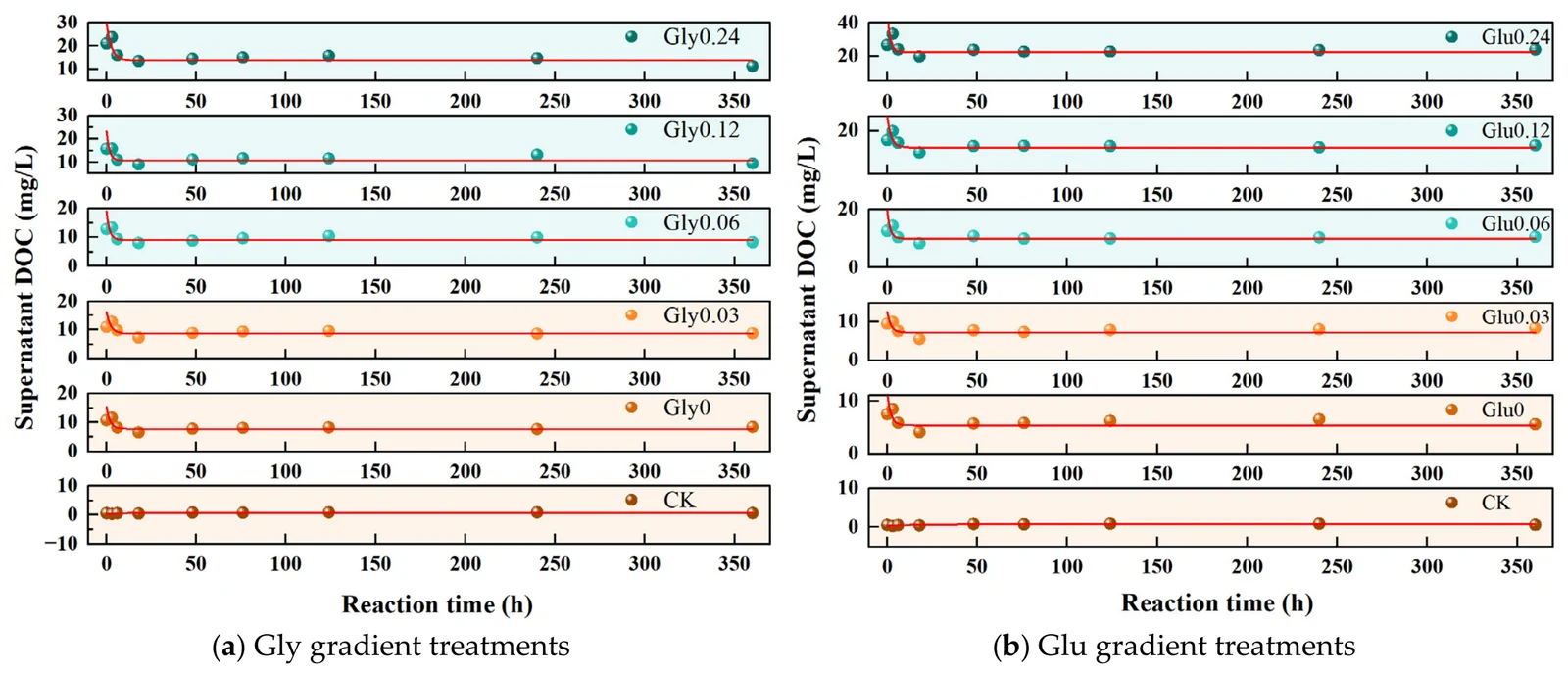
Temporal dynamics of supernatant DOC under different Gly (**a**) and Glu (**b**) concentration gradients.

As shown in Figure 2 and Table 2, parameter *a* (the asymptotic maximum stable DOC content) exhibited consistent increasing trends in both the Gly and Glu series. Parameter *a* in the Gly series increased from 7.7517 to 13.79955 mg/L (a 78.02% increase), while that in the Glu series rose from 5.34777 to 22.60267 mg/L (a 322.66% increase). For parameter *b*, consistently negative values were observed across all treatments, which is an inherent mathematical feature of the asymptotic growth model. The absolute value of *b* reflects the total amplitude of DOC accumulation. Parameter *b* in the Gly series decreased consistently from −7.58241 to −15.99878, representing a 111.00% increase in absolute magnitude. The Glu series exhibited milder fluctuations but maintained an overall downward trend, with the absolute value of *b* increasing from 6.16129 to 24.22545 (*a* 293.19% increase).

### 2.2. C Content of Humic-like Acid (C_HLA_), Ratio of C Content in Humic-like Acid to That in Fulvic-like Acid (C_HLA_/C_FLA_ Ratio), and HLA E_4_/E_6_ Ratio

**Figure 3:**
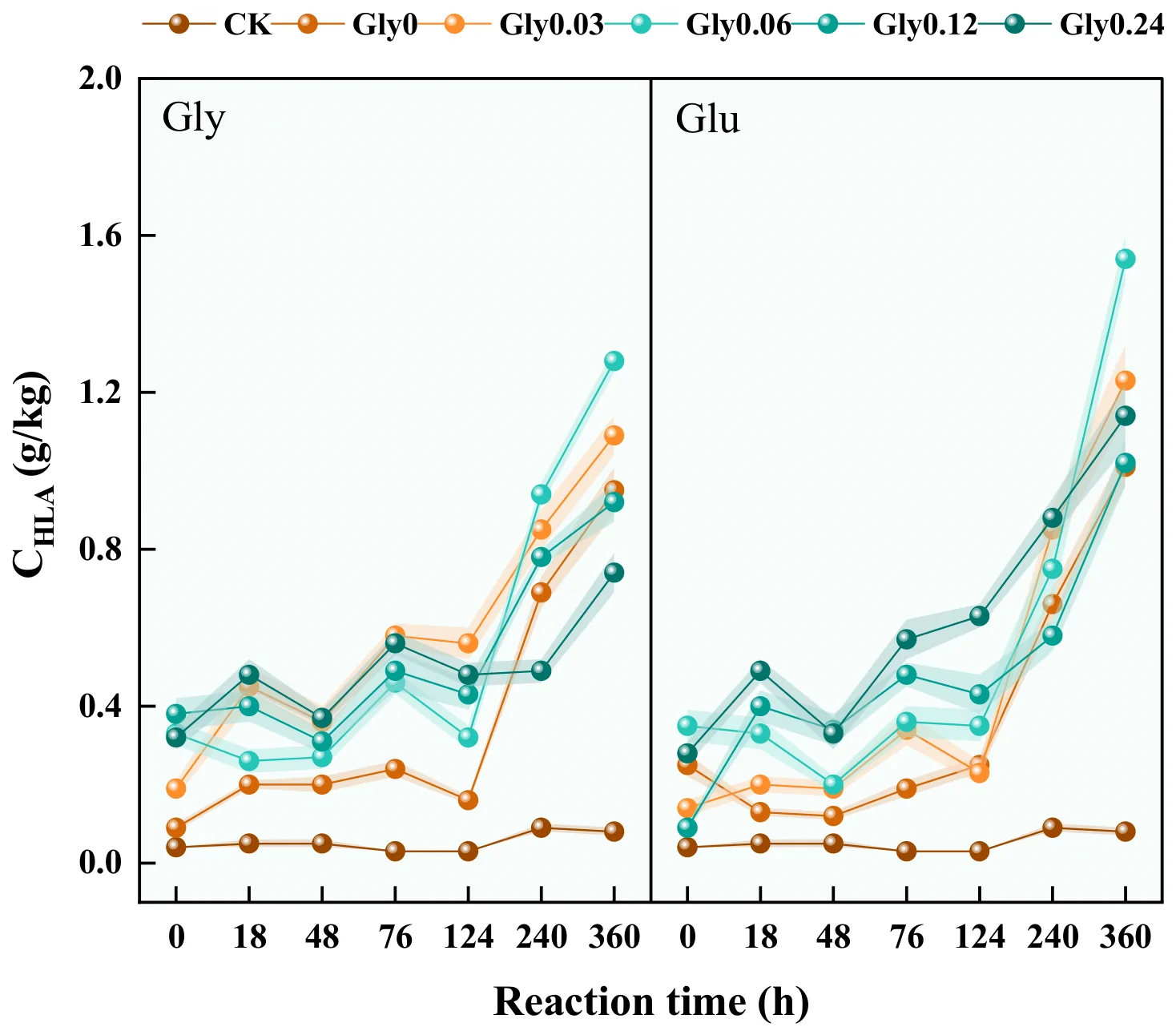
Accumulation dynamics of C_HLA_ under different Gly and Glu concentration gradients.

As shown in Figure 3, C_HLA_ exhibited pronounced temporal dynamics across all treatments. Specifically, in the early reaction stage (0–76 h), C_HLA_ fluctuated without sustained growth in both the Gly and Glu gradient series. In the late reaction stage (124–360 h), by contrast, C_HLA_ accumulation entered an accelerated phase in all treatments. For the Gly gradient series (Gly0 to Gly0.24), the average C_HLA_ contents over the entire incubation period were 0.38, 0.58, 0.55, 0.53, and 0.49 mg/L, respectively. The Gly series exhibited a typical dose–response pattern, with the Gly0.03 treatment yielding the highest C_HLA_ concentration. Consistently, the C_HLA_ of the Gly0 treatment was significantly lower than that of all other Gly-amended treatments. In contrast, the average C_HLA_ values for the Glu gradient series (Glu0 to Glu0.24) were 0.46, 0.45, 0.48, 0.48, and 0.49 mg/L, respectively. Notably, the C_HLA_ of the Glu0 treatment was significantly higher than that of the Gly0 treatment.

**Figure 4:**
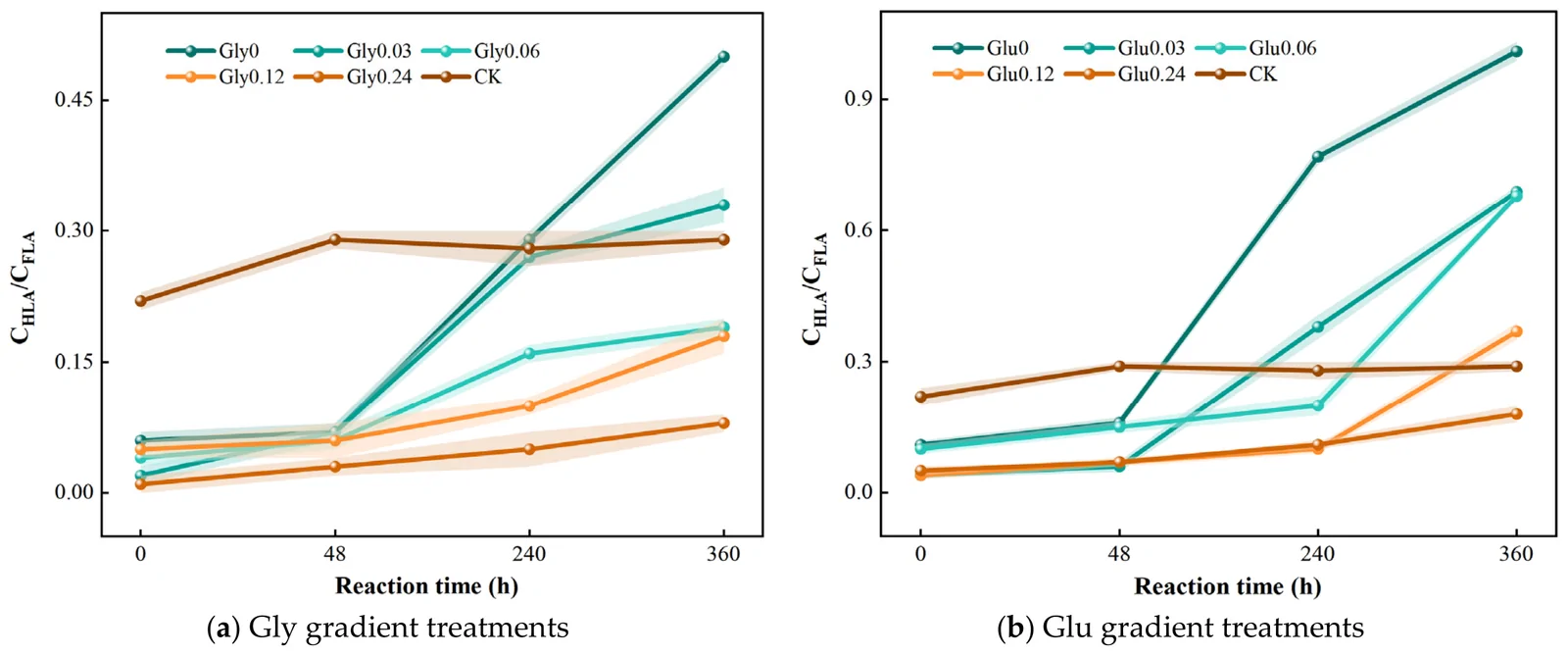
Temporal variations in the C_HLA_/C_FLA_ ratio under different Gly (**a**) and Glu (**b**) concentration gradients.

The C_HLA_/C_FLA_ ratio is a critical indicator for evaluating the humification degree and maturation level of HLS [17]. As shown in Figure 4, the C_HLA_/C_FLA_ ratio increased continuously with prolonged incubation time across all Gly and Glu treatments. Concentration-dependent regulatory effects were further observed for both precursors. For the Gly series, the mean C_HLA_/C_FLA_ ratio over the entire incubation period decreased significantly from 0.23 to 0.04 with rising Gly concentration. An analogous concentration-dependent reduction was observed in the Glu series, with the average C_HLA_/C_FLA_ ratio decreasing from 0.51 to 0.10 with rising Glu concentration.

**Figure 5:**
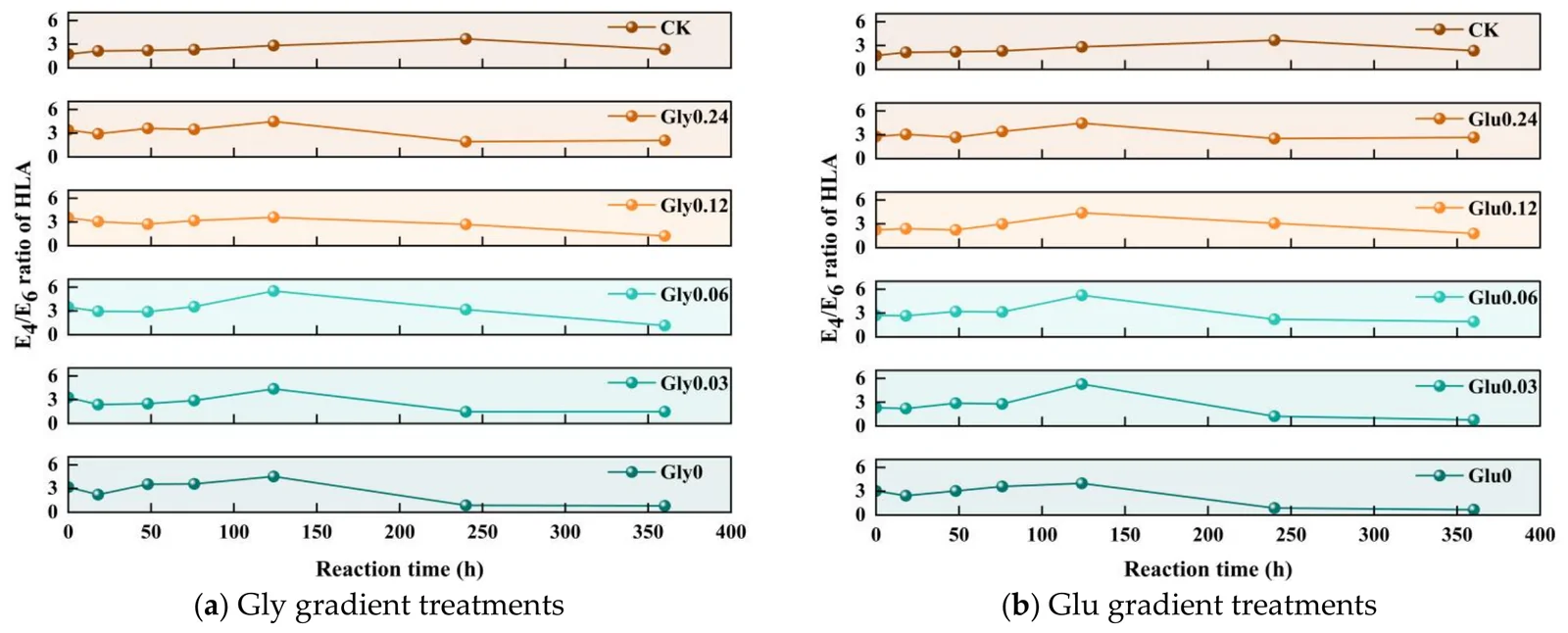
Temporal variations in the E_4_/E_6_ ratio of HLA extracted from goethite-mediated dark-brown residues under different Gly (**a**) and Glu (**b**) concentration gradients.

The E_4_/E_6_ ratio of HLA exhibited distinct dynamic changes throughout the reaction process, and this ratio is negatively correlated with the humification degree of HLS [17,18]. As shown in Figure 5, the E_4_/E_6_ ratio increased rapidly in the initial reaction stage, peaked at 76 h, and then declined gradually across all treatments.

Compared with the initial value at 0 h, the E_4_/E_6_ ratio of HLA at 360 h decreased by 73.94%, 54.54%, 66.61%, 64.74%, and 38.78% for the Gly0, Gly0.03, Gly0.06, Gly0.12, and Gly0.24 treatments, respectively. For the Glu gradient series, the E_4_/E_6_ ratio of HLA at 360 h decreased by 77.3%, 66.7%, 29.1%, 20.2%, and 4.0% relative to the initial value for the Glu0, Glu0.03, Glu0.06, Glu0.12, and Glu0.24 treatments, respectively. Overall, the magnitude of the E_4_/E_6_ ratio reduction decreased gradually with rising Gly and Glu concentrations.

### 2.3. Atomic Molar Ratios of HLA and FTIR Spectra of Dark-Brown Residues

To elucidate the elemental structural characteristics of HLA, atomic molar ratios (H/C, C/N, O/C) were calculated based on the mass fractions of C, H, O, and N obtained from elemental analysis. The molar ratio of a target element to carbon is defined as the ratio of their respective molar amounts, with the general formula as follows (Equation (3)):

(3) $$\frac{X}{C}\;=\;\frac{w_{X}\;/\;M_{X}}{w_{C}\;/{\;M}_{C}}$$

where *X* denotes the target element (H, N, or O); *w_X_* and *w_C_* represent the mass fractions of element *X* and *C* in HLA, respectively; *M_X_* and *M_C_* are the relative atomic masses of the corresponding elements (*M*_C_ = 12.01 g/mol, *M*_H_ = 1.008 g/mol, *M*_N_ = 14.007 g/mol, and *M*_O_ = 15.999 g/mol).

As shown in Table 3, the H/C ratio is negatively correlated with aromaticity, with lower values indicating a higher degree of aromatic condensation [18]. The C/N ratio reflects the relative abundance of C and N in HLA molecules; a decrease in the C/N ratio indicates preferential enrichment of N within stable, N-rich structural domains [18]. The O/C ratio serves as an indicator of oxidation degree and molecular polarity, with higher values corresponding to higher oxidation levels and greater molecular polarity [18]. The H/C ratios of both the Gly and Glu series exhibited increasing trends with rising substrate concentration. For the C/N ratio, a gradual decreasing trend was observed in the Gly series with increasing concentration, whereas the C/N ratio of the Glu series showed the opposite increasing trend. For the O/C ratio, both the Gly and Glu series exhibited increasing trends with rising reactant concentrations.

To semi-quantitatively evaluate the relative abundance of functional groups and mineral–organic interaction sites in dark-brown residues, peak deconvolution of FTIR spectra was performed, and the relative integrated area of each characteristic absorption band was calculated. The relative proportion of a single peak is defined as the percentage of its integrated area relative to the total integrated area of all selected characteristic peaks, calculated as follows (Equation (4)):

(4) $$R_{i}=\frac{A_{i}}{\sum_{i=1}^{n}A_{i}}\times \;100\%$$

where *R_i_* is the relative area percentage of the *i*-th characteristic peak; *A_i_* is the integrated peak area of the corresponding absorption band after curve fitting; *n* is the total number of selected characteristic peaks (*n* = 4 in this study, corresponding to 3426, 1630, 890, and 790 cm^−1^).

**Figure 6:**
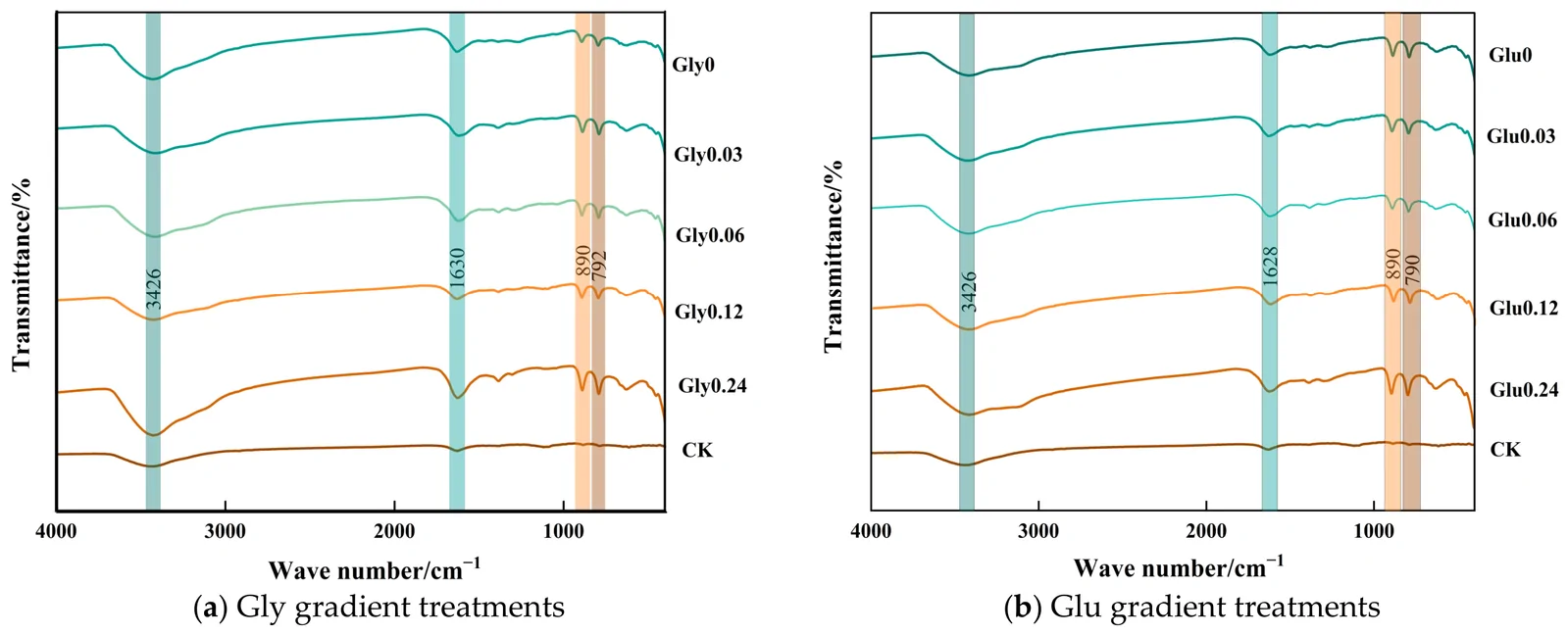
FTIR spectra of dark-brown residues obtained under Gly (**a**) and Glu (**b**) concentration gradients.

Four characteristic absorption peaks at 3426, 1630, 890, and 790 cm^−1^ were analyzed to investigate structural changes in dark-brown residues across the reaction system (Figure 6 and Table 4). The broad absorption band at 3426 cm^−1^, attributed to the stretching vibration of –OH groups (including phenolic hydroxyl, aliphatic hydroxyl, and hydrogen-bonded adsorbed water), exhibited divergent trends between the Gly and Glu series [19]. Specifically, the relative peak area percentage in the Gly series decreased progressively with increasing reactant concentration, whereas the Glu series exhibited a continuous increase in peak area percentage with rising Glu concentration.

The absorption peak at 1630 cm^−1^, corresponding to overlapping vibrations of aromatic C=C stretching and adsorbed water H–O–H bending, also exhibited concentration-dependent variations [20,21]. In the Gly series, the relative peak area percentage increased monotonically with rising Gly concentration. In contrast, the Glu series exhibited a biphasic response: the peak area percentage first increased with Glu concentration, reached a maximum of 21.9% at the Glu0.06 treatment, and then declined at higher Glu concentrations. For the iron-related characteristic peaks, the absorption band at 890 cm^−1^ (assigned to surface Fe–OH groups) and the peak at 790 cm^−1^ (attributed to Fe–O–Fe stretching vibrations in the iron oxide lattice) both showed consistent patterns across all treatments [22,23]. The relative peak area percentages of these two bands in the CK group were significantly lower than those in all experimental groups with Gly or Glu addition.

### 2.4. FTIR Spectra of Reacted Goethite

**Figure 7:**
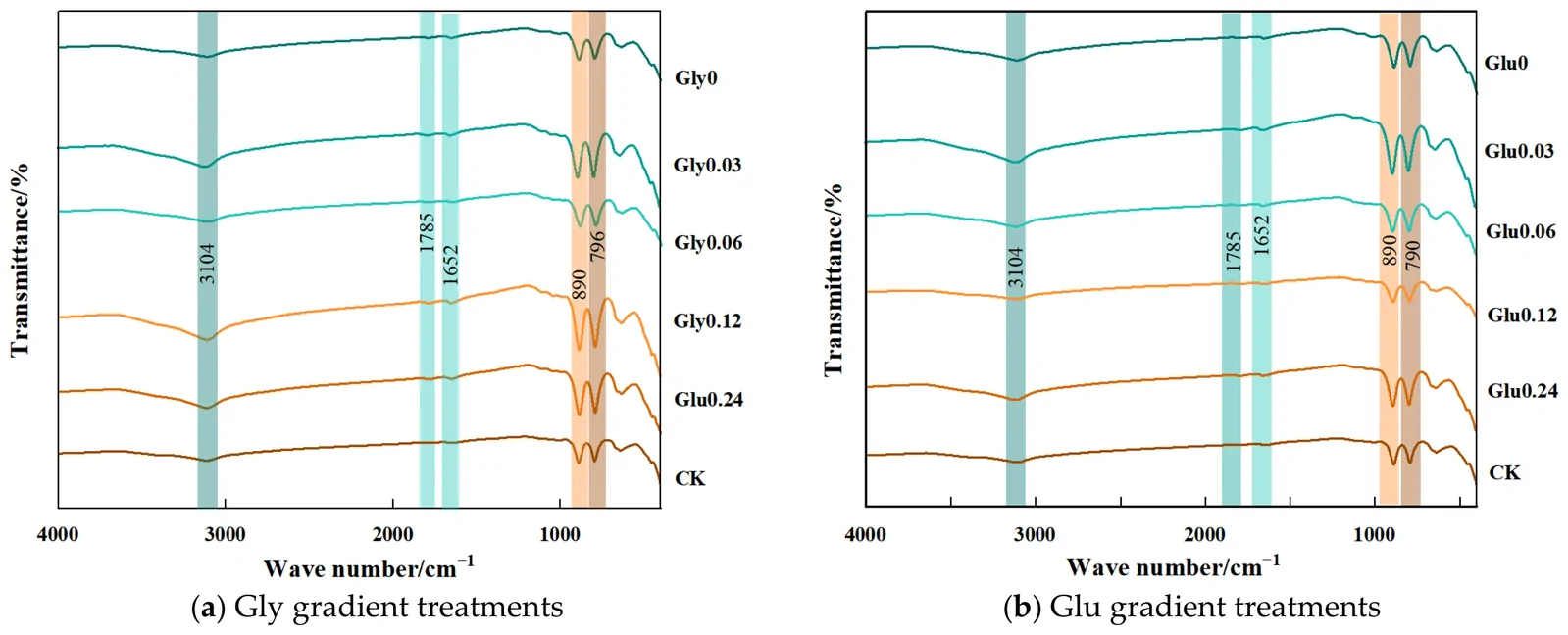
FTIR spectra of reacted goethite under Gly (**a**) and Glu (**b**) concentration gradients.

It should be noted that reacted goethite samples were cleaned with 30% H_2_O_2_ before FTIR characterization, and this strong oxidative treatment may alter the surface redox state and hydroxylation degree. The following results reflect the surface characteristics of cleaned goethite rather than the in-situ reaction interface. Bands at 3104 cm^−1^ correspond to stretching vibrations of structural hydroxyl groups (≡Fe–OH); 1785 cm^−1^ to Fe–O–H bending vibration; 1652 cm^−1^ to adsorbed water H–O–H bending; 890 cm^−1^ to bending vibrations of surface Fe–OH; and 796 cm^−1^ to Fe–O–Fe lattice vibrations [22,23,24,25,26]. As shown in Figure 7 and Table 5, the intensity of the 3104 cm^−1^ absorption peak in the Gly series exhibited a unimodal trend (increasing first and then decreasing) with rising Gly concentration. Notably, the relative peak area percentage of the CK group was significantly higher than that of all experimental groups. At 1785 cm^−1^, corresponding to Fe–O–H groups, the relative peak area percentage of the CK group was much lower than that of all treatment groups. A similar pattern was also observed at 1652 cm^−1^, which represented the peak of adsorbed water H–O–H bending. For the mineral-related absorption peaks at 890 cm^−1^ and 796 cm^−1^ related to goethite Fe–OH and Fe–O–Fe, peak intensities in the CK group were lower than those in all treatment groups.

### 2.5. PLS-SEM Pathway Analysis

**Figure 8:**
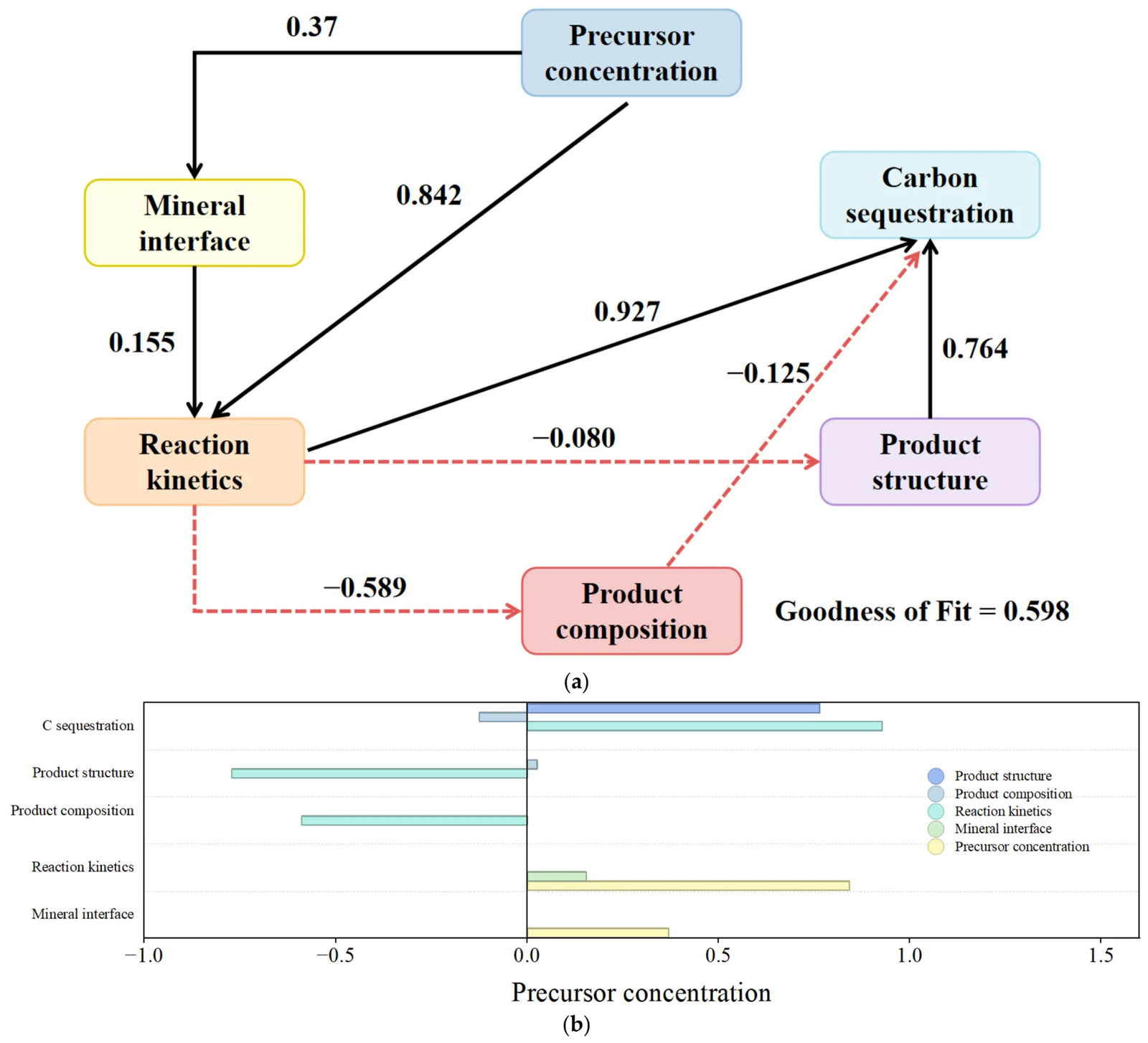
PLS-SEM model of C sequestration regulatory pathways (**a**) and total effects of precursor concentration on each latent variable (**b**) in goethite-mediated humification. (**a**) Structural equation model quantifying the multi-pathway regulatory network of C sequestration in goethite-mediated abiotic humification. (**b**) Magnitude and direction of total effects exerted by precursor concentration on intermediate processes and final C sequestration outcomes.

To avoid self-correlation between latent variables and ensure the independence of kinetic indicators and C sequestration outcomes, the PLS-SEM model was reconstructed with fully independent observed indicators. The latent variables and their corresponding observed indicators of the PLS-SEM model are listed in Table 6. PLS-SEM identified four positive causal pathways (Figure 8). Precursor concentration exerted the strongest positive effect on reaction kinetics (standardized path coefficient = 0.842), verifying that substrate availability is the dominant driver of humification reaction rates. A one standard deviation increase in precursor concentration corresponded to a 0.900 standard deviation increase in the reaction kinetics index. This result is consistent with the experimental observation that DOC accumulation in the Glu series increased significantly as concentration rose from 0 to 0.24 mol/L. Product structure also exhibited a significant positive effect on carbon sequestration efficiency (path coefficient = 0.764). Reaction kinetics exerted a significant positive effect on C sequestration efficiency (path coefficient = 0.927). Product structure also exhibited a significant positive effect on C sequestration efficiency (path coefficient = 0.764). The goodness-of-fit (GoF) of the PLS-SEM model was 0.598, indicating a good model fit with strong explanatory power for the C sequestration regulatory pathway. All path coefficients were statistically significant (*p* < 0.05) based on 1000 bootstrap resampling iterations.

## 3. Discussion

### 3.1. Effects of Gly and Glu Concentrations on Supernatant E_4_/E_6_ Ratio and DOC

The parameter *y*_0_, which reflects the baseline aromaticity of nascent HLS, increased sharply by 108.87% with increasing Gly but only slightly by 20.38% with increasing Glu concentration. This pattern was consistent with previous findings in gibbsite-catalyzed humification systems, where Gly also exerted a stronger effect on HLS aromaticity than Glu [13,17]. However, the magnitude of aromaticity enhancement in the present goethite system was approximately 1.6-fold higher than that reported for the gibbsite system, which could be attributed to the stronger oxidative catalytic activity of Fe(III) redox sites on goethite that accelerated heterocyclic aromatic formation. Unlike prior studies that only reported overall humification yield under fixed C/N ratios [15], this study further quantified the independent contributions of N and C precursors to kinetic parameters across a full concentration gradient. This divergence stemmed from distinct mineral interfacial behaviors and functional roles of the two precursors in the presence of goethite. Gly’s amino groups formed strong H-bonds and underwent ligand exchange with goethite’s abundant surface hydroxyls, which drove interfacial enrichment [27,28]. This process was proposed to accelerate Maillard nucleophilic addition between amino and carbonyl groups and directly form N-containing aromatic heterocycles—key structures that govern HLS aromaticity [29]. Notably, the current experimental design does not include a substrate-only control without goethite. The rationale for omitting this control is that the Maillard reaction cannot proceed smoothly in the absence of goethite: even with the addition of the three Maillard reaction precursors, no reaction products will be formed, and accordingly no relevant indicators can be measured. This has been clearly confirmed in preliminary experiments. As a result, we cannot fully disentangle goethite-catalyzed reactions from spontaneous Maillard polymerization and catechol oxidation. The observed reaction enhancements should be interpreted as combined effects of mineral-mediated processes and spontaneous abiotic humification. In contrast, Glu primarily underwent ring-opening and dehydration on the mineral surface to supply aliphatic C skeletons. It lacked aromatic cyclization-active groups and barely contributed to aromatic nucleus formation, resulting in negligible impacts on baseline aromaticity [30]. Thus, N-precursors dominated initial aromaticity via interfacial heterocycle synthesis, while C-precursors acted only as structural scaffolds. The unimodal trend of *x_c_* observed in both Gly and Glu treatments mirrored the balance between precursor availability and goethite’s catalytic capacity. At low concentrations, abundant unsaturated ≡Fe–OH sites rapidly adsorbed and activated precursors, synchronized the Maillard and polyphenol oxidation pathways to accelerate intermediate turnover, and reduced *x_c_* [31]. Moderate concentrations optimized the C/N stoichiometry, matched the rates of amino-carbonyl condensation and oxidative polymerization, achieved the maximum pathway coupling efficiency, and minimized *x_c_* [29]. Excessive precursors triggered intense competitive adsorption and steric hindrance, blocked interfacial electron transfer, slowed intermediate transformation, and delayed *x_c_* [32]. This finding confirmed that concentration modulated humification kinetics by regulating interfacial adsorption competition and pathway synergy. Similarly, for both precursors, the minimal *w* at 0.12 mol/L reflected optimal substrate–catalyst matching. At this concentration, precursor diffusion flux aligned with goethite’s catalytic turnover rate and maintained a dynamic equilibrium between intermediate generation and transformation without accumulation or side reactions. Deviations disrupted this balance: low concentrations left active sites underutilized and caused intermittent reactions; high concentrations saturated active sites and induced precursor self-polymerization, resulting in a prolonged active period and reduced reaction efficiency. Parameter *a* denotes the asymptotic maximum stable DOC content and represents the potential for stable dissolved humic-like substance generation in the goethite-catalyzed abiotic humification system. Both Gly and Glu treatments elicited a consistent increase in *a*. Gly elevated *a* by 78.02%, while Glu drove a far more substantial increase of 322.66%. It should be emphasized that supernatant DOC cannot represent total carbon retention in the system, as it does not account for mineral-adsorbed carbon, insoluble precipitates and CO_2_ mineralization; thus, it cannot be directly equated to C sequestration capacity. This divergent capacity enhancement was governed by precursor-specific C stoichiometry and mineral-interface functional mechanisms. As a hexose (C_6_H_12_O_6_), Glu provided a high molar C load and acted as the primary aliphatic C skeleton donor for HLS biosynthesis [33]. At the goethite interface, abundant surface hydroxyl groups concentrated Glu via hydrogen bonding and triggered sequential sugar ring opening, dehydration, and polymerization. These reactions generated continuous aliphatic C chains that served as the structural backbone for HLS and directly expanded the system’s C sequestration capacity [34].

The consistently negative *b* values are an inherent mathematical property of the first-order asymptotic growth model, reflecting that initial DOC concentrations are lower than the final asymptotic plateau. The absolute value of *b* reflects the dynamic amplitude of net DOC accumulation toward the asymptotic maximum and characterizes the kinetic efficiency of labile-to-recalcitrant C transformation under goethite mediation [26]. The increasing absolute magnitude of *b* across concentration gradients confirmed net sequestration of stable organic C through the conversion of soluble low-molecular-weight precursors into mineral-associated organic matter (MAOM) and high-molecular-weight HLS [35,36]. In contrast, the CK control exhibited a positive *b* value (+0.49174). This is because the blank system contained no exogenous organic precursors, and trace organic impurities leached from the goethite crystal lattice led to a gradual increase in supernatant DOC over time, resulting in an ascending asymptotic kinetic pattern. This distinct kinetic behavior further verified that the DOC decline in precursor-amended treatments was driven by humification polymerization and mineral adsorption rather than system noise.

The magnitude of change in *b* differed markedly between treatments. Gly induced a 111.00% increase in magnitude, while Glu triggered a 293.19% rise in magnitude. Such discrepancies were driven by distinct kinetic regulatory mechanisms of C supply and N-mediated interfacial interactions. For Glu treatments, sufficient C input sustained a persistent flux of aliphatic intermediates at the goethite surface and maintained high catalytic turnover rates for oxidative polymerization [30,37]. This continuous C substrate supply eliminated kinetic limitations, enabled efficient conversion of soluble organic C into stable MAOM and HLS, and thus amplified the amplitude of net DOC accumulation. For Gly treatments, N modulated humification kinetics rather than C transformation flux [30,36]. Gly accelerated aromatic core formation and intermolecular cross-linking of HLS, optimized reaction kinetics, but lacked abundant C substrates to drive large-scale C conversion. At high Gly concentrations, excess amino groups competed with organic intermediates for active hydroxyl sites on goethite, hindered interfacial electron transfer, and suppressed oxidative polymerization. This competitive adsorption subtly constrained the rate of labile C conversion and resulted in a smaller change in *b* relative to Glu treatments [27,28].

### 3.2. C_HLA_, C_HLA_/C_FLA_ Ratio, and E_4_/E_6_ Ratio of HLA

The fluctuating changes in C_HLA_ without sustained growth during the early stage (0–76 h) were attributed to the dynamic equilibrium of adsorption–desorption and the transient nature of low-molecular-weight intermediates at the goethite interface. ≡Fe–OH reversibly adsorbed Cat, Glu, and Gly via hydrogen bonding and ligand exchange [38]. Upon adsorption, early-stage reactions were dominated by the generation of small, weakly aromatic intermediates that exhibited weak binding affinity for goethite surfaces and were prone to desorption into the aqueous phase or reversible structural rearrangement, thereby resulting in the unstable fluctuation of C_HLA_ (Equation (5)).

(5) $$\mathrm{Weakly}\;\mathrm{aromatic}\;\mathrm{intermediates}\overset{\;\equiv Fe^{3+}}{⟶}\;\mathrm{HLA}(\mathrm{Ar}–\mathrm{OH},–\mathrm{COOH})\overset{\equiv \mathrm{Fe}–\mathrm{OH}}{⟶}\equiv \mathrm{Fe}–\mathrm{OOC}–\mathrm{HLA}+H_{2}O$$

As labile precursors were depleted, intermediates underwent oxidative polymerization and cross-linking to form HLA enriched in aromatic and polar functional groups (Equation (6)). These functional groups form strong coordination bonds with surface Fe^3+^, enabling irreversible organic carbon immobilization and accelerated C_HLA_ accumulation [39]. For the Gly series, the significant dose–response relationship highlighted the critical regulatory role of nitrogen in HLA structural maturation. Moderate Gly concentrations (0.03 mol/L) drove nucleophilic condensation and oxidative coupling, forming aromatic heterocycles and amide linkages that enhanced HLA stability.

(6) $$\mathrm{Glu}–\mathrm{CHO}+\mathrm{Gly}–NH_{2}+\mathrm{Quinone}\overset{\equiv \mathrm{Fe}–\mathrm{OH}/Fe^{3+}}{⟶\;}\mathrm{Ar}–\mathrm{CONH}–\mathrm{HLA}+2H_{2}O$$

This optimal stoichiometry maximized HLA aromaticity and mineral-binding affinity, leading to the highest C_HLA_ in the Gly0.03 treatment. Excess Gly disrupted this balance via competitive adsorption: surplus amino groups occupied goethite active sites, inhibited aromatic polymerization, and reduced HLA yield and stability, thereby lowering C_HLA_ [27,28]. The significantly lower C_HLA_ in the Gly0 treatment confirmed that N deficiency severely restricted the formation of stable HLA (Equation (7)). In the absence of Gly, humification proceeded solely via the polyphenol oxidation pathway, which generated HLA with low aromaticity and weak mineral-binding capacity:

(7) $$\mathrm{nCat}\overset{\equiv Fe^{3+}}{⟶}\mathrm{Low-aromatic}\;\mathrm{HLA}\;+\;nH_{2}O$$

In contrast to the Gly series, the relatively stable C_HLA_ across the Glu concentration gradient indicated that Glu primarily acted as an aliphatic skeleton donor rather than a key regulator of HLA–mineral interactions. Glu provided essential aliphatic C backbones for HLA construction via ring-opening, dehydration, and polymerization reactions at the goethite interface [30,34]. Within the tested concentration range, the supply of aliphatic C skeletons reached a saturated state, leading to negligible impacts of Glu concentration variations on HLA aromaticity, functional group composition, and mineral-binding affinity, and thus resulting in non-significant differences in C_HLA_ among Glu treatments. Notably, the higher C_HLA_ in the Glu0 treatment compared with the Gly0 treatment reflected divergent humification pathways under N-free conditions: the Glu0 treatment favored direct oxidative polymerization of Cat on goethite surfaces to generate moderately aromatic HLA with stable mineral-binding ability, while the Gly0 treatment lacked N to sustain stable cross-linking of humification intermediates, leading to the formation of labile, easily desorbed low-aromatic products [40]. These findings collectively demonstrated that N (supplied by Gly) was indispensable for enhancing the structural stability and mineral immobilization potential of humified products, while C precursors (supplied by Glu) form the fundamental material basis for organic c sequestration in iron-rich soil environments [36,41,42]. Notably, the above differences were jointly driven by two mechanisms: the distinct structural roles of N and C precursors, and the compound-specific sorption affinity of Gly and Glu on goethite surfaces. The difference in adsorption strength led to differentiated enrichment efficiency of precursors at the reaction interface, which further amplified the divergence in reaction pathways and product composition.

In this study, a continuous temporal increase in the C_HLA_/C_FLA_ ratio was universally observed across all Gly and Glu treatments, providing direct evidence for progressive HLS maturation driven by goethite-mediated interfacial catalysis. The progressive increase in the C_HLA_/C_FLA_ ratio over incubation time aligned with the general maturation trend observed in *δ*-MnO_2_- and gibbsite-mediated humification systems [15,17]. However, the inhibitory effect of excessive precursors on humification maturity was more pronounced in the goethite system than in the gibbsite system, likely due to the higher surface reactivity of goethite that led to stronger competitive adsorption at high precursor loadings. This consistent trend was mechanistically underpinned by the sequential activation and transformation of FLA intermediates at the goethite–water interface. Specifically, the abundant surface ≡Fe–OH and Fe(III) redox centers of goethite first adsorb and activate FLA molecules via hydrogen bonding and ligand exchange [38]. These activated species then underwent three interconnected catalytic pathways: oxidative polymerization of phenolic moieties, dehydration condensation of sugar-derived carbonyl groups, and intermolecular cross-linking of reactive functional groups [43,44,45]. Collectively, these reactions assembled small, labile FLA molecules into large, structurally complex HLA macromolecules with condensed aromatic cores and rigid cross-linked networks, driving the steady elevation of the C_HLA_/C_FLA_ ratio throughout the incubation period.

For Gly-amended treatments, the mean C_HLA_/C_FLA_ ratio decreased significantly from 0.23 (Gly0) to 0.04 (Gly0.24) with rising Gly concentration, revealing a N-dependent regulatory mechanism mediated by goethite interfacial adsorption competition and reaction pathway partitioning [46]. At low Gly concentrations (0–0.03 mol/L), Gly functioned as an efficient amino donor that accelerated the Maillard reaction between its amino groups and carbonyl groups derived from Glu oxidation and Cat degradation [27,28]. This reaction formed stable amide bonds and N-containing aromatic heterocycles, which acted as molecular bridges to promote the cross-linking of FLA intermediates into HLA macromolecules, thereby enhancing HLA maturation. However, as Gly concentration exceeds 0.06 mol/L, excess Gly molecules triggered severe competitive adsorption for the limited ≡Fe–OH active sites on goethite surfaces [38]. The preferential adsorption of surplus amino groups blocked access of FLA intermediates and Cat molecules to catalytic Fe(III) centers, and inhibited the oxidative polymerization and cross-linking reactions required for FLA-to-HLA conversion [27,28]. Concomitantly, high Gly concentrations shifted the reaction pathway toward the formation of low-molecular-weight N-containing aliphatic compounds rather than large aromatic HLA macromolecules, as corroborated by the increasing H/C ratio and decreasing aromaticity of HLA observed in elemental analysis and FTIR spectroscopy [16]. This combination of inhibited FLA conversion and enhanced small-molecule production leads to the relative accumulation of FLA and results in a significant reduction in the mean C_HLA_/C_FLA_ ratio. An analogous concentration-dependent reduction in the mean C_HLA_/C_FLA_ ratio was observed in the Glu-amended treatments, where the value dropped from 0.51 (Glu0) to 0.10 (Glu0.24) with rising Glu concentration. This trend was governed by C precursor-induced catalytic site saturation and substrate dilution effects, which were fundamentally distinct from the N-mediated mechanism in the Gly series. As the primary aliphatic C skeleton donor, Glu provided essential structural building blocks for HLS formation through ring-opening, dehydration, and polymerization reactions at the goethite interface [30,34]. At low to moderate Glu concentrations (0–0.06 mol/L), the supply of aliphatic C skeletons was balanced with the availability of Gly and Cat, supporting efficient coupling between the Maillard reaction and polyphenol oxidative polymerization pathways [30,31]. This optimal stoichiometric balance promoted the conversion of FLA to HLA and maintained a relatively high C_HLA_/C_FLA_ ratio.

However, at high Glu concentrations (>0.06 mol/L), the excess supply of aliphatic C induced two detrimental effects on HLS maturation [47]. First, surplus Glu molecules and their aliphatic intermediates competed with FLA and Cat for goethite active sites, inhibiting the oxidative condensation reactions that drove HLA formation. Second, excess aliphatic C induced a substrate dilution effect, reducing the intermolecular collision frequency between reactive FLA intermediates and hindering their cross-linking into large HLA macromolecules. Furthermore, under fixed Gly concentration conditions, high Glu concentrations elevated the C/N ratio, which limited Maillard reaction capacity and led to the accumulation of aliphatic FLA intermediates rather than aromatic HLA. These combined effects explained the progressive decrease in the mean C_HLA_/C_FLA_ ratio with rising Glu concentration [48].

Notably, the mean C_HLA_/C_FLA_ ratio in the Glu0 treatment (0.51) was more than twice that in the Gly0 treatment (0.23), highlighting divergent humification pathways under nitrogen-deficient conditions. In the absence of Gly (Gly0), humification proceeded solely via the polyphenol oxidation pathway, which generated HLA with low aromaticity and weak mineral-binding affinity, leading to higher FLA accumulation and a lower C_HLA_/C_FLA_ ratio. In contrast, in the Glu0 treatment, humification proceeds via coupled polyphenol oxidation and amino–carbonyl condensation pathways, generating more stable HLA with higher mineral-binding capacity and resulting in a higher C_HLA_/C_FLA_ ratio [31].

Across all treatments, the HLA E_4_/E_6_ ratio exhibited a consistent kinetic pattern governed by goethite-mediated interfacial catalysis: a rapid rise within 0–76 h, peaking at 76 h, followed by a gradual decline until 360 h. This dynamic directly reflected sequential precursor transformation at the mineral–water interface. In the early stage, ≡Fe–OH catalyzed Glu cleavage and partial Cat degradation, generating large amounts of low-molecular-weight, weakly aromatic aliphatic/phenolic intermediates [38]. Their accumulation drove the E_4_/E_6_ rise, and the 76 h peak marked the maximum buildup of immature intermediates and the transition to condensation polymerization. After 76 h, surface Fe^3+^/Fe^2+^ redox couples triggered electron transfer and catalyzed oxidative polymerization, aromatic ring condensation, and intramolecular cross-linking [49]. These reactions converted low-aromatic precursors into high-molecular-weight HLA with condensed aromatic cores, reducing the E_4_/E_6_ ratio and enhancing the structural stability of HLS. For Gly-amended treatments, the amplitude of E_4_/E_6_ reduction progressively diminished from 73.94% (Gly0) to 38.78% (Gly0.24) with rising Gly concentration, revealing an N-dependent interfacial regulatory mechanism for aromatic condensation. Under N limitation (Gly0, Gly0.03), the absence of amino groups restricted the Maillard reaction, and humification was dominated by polyphenol oxidation. Goethite selectively adsorbed Cat via hydrogen bonding, and Fe^3+^-catalyzed oxidation produced quinone intermediates that self-condense into highly aromatic structures, driving a substantial E_4_/E_6_ reduction [50]. Elevated Gly introduced amino groups that competed with phenolics for ≡Fe–OH sites via ligand exchange, blocked Cat access to catalytic Fe^3+^ sites, and suppressed aromatic condensation. Meanwhile, the enhanced Maillard reaction inserted aliphatic amide moieties into HLA backbones, diluted aromatic core density, and attenuated the E_4_/E_6_ decline.

In Glu-amended treatments, the reduction amplitude plummeted from 77.3% (Glu0) to 4.0% (Glu0.24), uncovering a C-skeleton-driven mechanism that inhibited aromatic maturation. Glu acted as the primary aliphatic C donor, and low concentrations (Glu0–Glu0.06) supported polyphenol oxidation and robust aromatic condensation. Excess Glu underwent ring-opening and dehydration at the goethite interface, generating abundant aliphatic aldehydes [30,34]. These intermediates preferentially underwent the Maillard reaction, extensively inserting aliphatic chains into HLA aromatic backbones to disrupt conjugated π-electron systems and reduce aromaticity. Additionally, surplus aliphatic intermediates occupied active catalytic sites and inhibited Cat adsorption and oxidative condensation. At high Glu concentrations, aliphatic C dominates, nearly counteracting aromatic condensation and limiting humification maturation [13,17].

### 3.3. Elemental Molar Ratios of HLA and FTIR Spectra of Dark-Brown Residue

In both the Gly and Glu series, H/C ratios exhibited consistent increasing trends with rising precursor concentrations, indicating a progressive shift toward more aliphatic-dominated HLA structures at higher substrate loadings. However, the magnitude of this increase differed markedly between the two series, with the Gly series showing a substantially larger increment than the Glu series, reflecting precursor-specific structural modification pathways. For the Gly series, the pronounced elevation in H/C ratio arose from two interconnected interfacial catalytic processes. First, at high Gly concentrations, excess amino groups preferentially underwent nucleophilic addition with carbonyl intermediates derived from Glu oxidation and Cat degradation at goethite ≡Fe–OH active sites, forming abundant –CONH– rather than N-containing aromatic heterocycles [29]. Second, competitive adsorption of excess Gly molecules blocked Cat access to goethite Fe(III) redox centers, inhibiting the oxidative polymerization of polyphenols—the primary source of aromatic C skeletons in the integrated polyphenol–Maillard system. As a result, the relative proportion of aliphatic amide structures increased at the expense of condensed aromatic domains, leading to a significant rise in the H/C ratio [51,52]. In contrast, the moderate increase in the H/C ratio in the Glu series stemmed from excessive aliphatic C supply rather than a fundamental pathway shift. As the primary C source, Glu underwent sequential ring-opening, dehydration, and polymerization reactions at goethite–water interfaces, generating linear aliphatic chains with high hydrogen content [30,34]. At low to moderate Glu concentrations, these aliphatic intermediates were efficiently cross-linked into aromatic networks via Maillard reactions with Gly, maintaining a relatively balanced aromatic–aliphatic composition. At Glu concentrations exceeding 0.06 mol/L, however, the fixed Gly supply (0.06 mol/L) became rate-limiting for Maillard cross-linking, resulting in the accumulation of unreacted aliphatic intermediates and their partial incorporation into HLA structures. This reduced the overall aromatic density of HLA and led to a moderate increase in the H/C ratio.

Correspondingly, the diametrically opposite evolution of C/N ratios in the Gly and Glu series highlighted the distinct roles of these precursors in regulating elemental stoichiometry during abiotic humification. The C/N ratio provided critical insights into the relative incorporation of C and N into HLA molecules and reflected the partitioning of N between stable structural domains and labile aliphatic fractions. In the Gly series, C/N ratios decreased monotonically from 29.78 to 7.91 with rising Gly concentration, demonstrating progressive and preferential nitrogen enrichment in HLA structures governed by the strong interfacial affinity of Gly for goethite surfaces. Gly molecules were rapidly adsorbed onto goethite ≡Fe–OH sites via ligand exchange between their α-amino groups and surface hydroxyls, resulting in significant N accumulation at the mineral–water interface. This interfacial N pool then participated in two key reactions: nucleophilic addition with Glu-derived carbonyls to form amide linkages and Schiff bases, and oxidative coupling with Cat quinones to generate N-containing aromatic heterocycles. These N-rich moieties exhibited high chemical stability and strong binding affinity for goethite Fe(III) sites, leading to their preferential retention in the HLA fraction rather than partitioning into the more labile FLA fraction [27]. The continuous decrease in the C/N ratio even at the highest Gly concentration (0.24 mol/L) indicated that N incorporation into HLA was not saturated under the tested conditions, suggesting that goethite-mediated abiotic humification had substantial capacity for N sequestration in iron-rich soil environments [17,53,54].

For the Gly series, the increase in the O/C ratio was primarily attributed to amide bond formation and oxidative carbonyl generation rather than hydroxyl accumulation. High Gly concentrations promoted the formation of –CONH– groups, each contributing one O atom per incorporated N atom. Additionally, goethite-mediated oxidative deamination of excess Gly generated glyoxylic acid and other O-rich intermediates, which were further cross-linked into HLA structures. The substantial increase in the O/C ratio also indicated that N incorporation into HLA was accompanied by extensive oxidation, which enhanced the polarity of HLA molecules and their binding affinity for goethite surfaces via hydrogen bonding and ligand exchange [7]. In the Glu series, the increase in the O/C ratio was predominantly driven by the accumulation of aliphatic hydroxyl groups. Glu underwent ring-opening reactions at goethite surfaces to form linear polyols with multiple terminal hydroxyl groups, which were partially incorporated into HLA structures without complete oxidation or condensation. At high Glu concentrations, the limited Maillard reaction capacity prevented the conversion of these hydroxyl-rich aliphatic intermediates into more condensed structures. Additionally, goethite-mediated oxidation of Glu generated aldehyde and carboxyl groups, which further contributed to the increase in the O/C ratio [55]. The smaller magnitude of the O/C increase in the Glu series compared with the Gly series indicated that N incorporation was associated with more extensive oxidation than C skeleton addition, highlighting the critical role of N in regulating the redox state of humification products.

The broad absorption band at 3426 cm^−1^, assigned to the stretching vibration of –OH groups of HLA, exhibited fundamentally opposite concentration-dependent trends in the Gly and Glu series. This striking divergence originated from the distinct chemical properties and functional roles of the two precursors in the integrated polyphenol–Maillard reaction system. In the Gly series, the progressive decrease in 3426 cm^−1^ peak intensity with rising Gly concentration was attributed to two interconnected interfacial catalytic mechanisms. First, as the sole N donor in the system, Gly participated in rapid nucleophilic addition with carbonyl groups derived from Glu oxidation and Cat degradation via the Maillard reaction, forming –CONH– groups that consumed reactive hydroxyl groups in organic intermediates. This process was significantly accelerated by goethite surface Fe(III) active sites acting as electron acceptors. Second, elevated Gly concentrations enhanced the formation of N-containing aromatic heterocycles via interfacial catalytic condensation, which involved intramolecular dehydration and cross-linking that reduced free hydroxyl groups and increased molecular condensation. This interpretation was strongly supported by the elemental analysis results, which showed a continuous decrease in the C/N ratio and an increase in the H/C ratio with rising Gly concentration, indicating enhanced N incorporation and aliphatic amide moiety formation in HLA molecules. In contrast, the continuous increase in the 3426 cm^−1^ peak intensity with rising Glu concentration reflected the dominant role of Glu as an aliphatic C skeleton donor rather than a structural modifier. Glu underwent sequential ring-opening, dehydration, and polymerization reactions at goethite–water interfaces, generating abundant aliphatic intermediates with terminal hydroxyl groups [30,34]. At high Glu concentrations, the excess aliphatic C supply led to the incorporation of more hydroxyl-rich aliphatic chains into HLA structures rather than their complete conversion into aromatic rings or amide bonds. The limited availability of Gly (fixed at 0.06 mol/L) restricted Maillard reaction capacity, resulting in the accumulation of unreacted or partially reacted aliphatic hydroxyl-containing intermediates. This mechanism was further corroborated by the increasing C/N ratio in the Glu series, demonstrating that C accumulation outpaced N incorporation and led to a higher proportion of aliphatic moieties in HLA. Notably, N–H stretching bands of primary amino groups (~3300–3500 cm^−1^) were not separately quantified in this study, as they extensively overlapped with the broad and intense O–H stretching absorption centered at 3426 cm^−1^ in the measured spectral range, making independent deconvolution unreliable.

The absorption peak at 1630 cm^−1^, corresponding to overlapping vibrations of aromatic C=C stretching and adsorbed water H–O–H bending, provided direct molecular evidence for the differential effects of Gly and Glu on HLA aromaticity. The monotonic increase in 1630 cm^−1^ peak intensity with rising Gly concentration confirmed that Gly was a key positive regulator of aromatic structure formation in abiotic humification. Gly-derived amino groups accelerated the Maillard reaction to generate N-containing aromatic heterocycles that contributed significantly to the C=C stretching signal, and also acted as cross-linking agents between aromatic intermediates produced by polyphenol oxidation, promoting the condensation of small phenolic molecules into larger aromatic networks [7]. This mechanism was consistent with the kinetic analysis results, which showed that the baseline aromaticity parameter (*y*_0_) of newly formed HLS increased by 108.87% with rising Gly concentration, compared with only 20.38% in the Glu series. Collectively, these findings demonstrated that Gly continuously enhanced the aromatic character of HLA by facilitating both heterocyclic aromatic formation and intermolecular cross-linking. The biphasic response of the 1630 cm^−1^ peak in the Glu series revealed a concentration-dependent trade-off between aromatic condensation and aliphatic chain insertion. At low to moderate Glu concentrations (0–0.06 mol/L), Glu provided sufficient C skeletons for both the Maillard reaction and polyphenol oxidative polymerization, supporting aromatic structure formation and leading to increased peak intensity. The maximum relative peak area of 21.9% at the Glu0.06 treatment corresponded to the optimal C/N stoichiometric balance for pathway coupling efficiency, as evidenced by the minimum intermediate peak time (*x_c_*) in the kinetic analysis [30]. However, at higher Glu concentrations (>0.06 mol/L), excess aliphatic intermediates preferentially underwent Maillard reactions with limited Gly molecules, resulting in extensive insertion of aliphatic chains into HLA backbones. This diluted the density of aromatic C=C groups and disrupted conjugated π-electron systems, thereby reducing the relative intensity of the 1630 cm^−1^ peak [13,17].

The significantly higher intensities of the 890 cm^−1^ and 790 cm^−1^ peaks in all substrate-amended treatments compared with the CK group provided evidence for substrate-induced changes in goethite surface functional groups and enhanced mineral–organic interfacial interactions. The goethite surface reconstruction induced by organic precursors was consistent with previous observations in ferrihydrite–organic matter interaction systems [12,56], but this study further demonstrated that both amino acid and sugar precursors could trigger such surface activation, expanding the scope of the mineral–organic co-evolution hypothesis. These iron-related characteristic peaks were direct indicators of goethite surface reactivity and the formation of stable mineral–organic complexes. In the absence of organic precursors, goethite maintained a thermodynamically stable surface with relatively low reactivity, resulting in weak vibrational signals for both surface Fe–OH and lattice Fe–O–Fe groups. However, when organic precursors were present, they formed strong coordination complexes with surface Fe^3+^ sites via ligand exchange and hydrogen bonding, which altered the local chemical environment of surface iron atoms and modified the abundance of surface Fe–OH groups. Furthermore, the adsorbed organic matrix rigidified the mineral surface structure, strengthening Fe–O–Fe lattice vibrations by reducing the mobility of iron atoms in the crystal lattice. Notably, the consistent enhancement of these iron-related peaks across all Gly and Glu concentrations indicated that both N and C precursors could modulate goethite surface reactivity, which was a prerequisite for efficient abiotic humification catalysis in the presence of goethite. However, FTIR data alone cannot directly confirm crystal lattice reconstruction or dissolution-recrystallization processes; further structural characterization is needed to verify these mechanisms. This surface activation mechanism explained the accelerated reaction kinetics observed in all substrate treatments compared with the CK group, as the exposed Fe–OH groups and Fe^3+^ active sites served as both adsorption sites for organic precursors and electron acceptors for oxidative polymerization reactions [56,57].

### 3.4. FTIR Functional Group Characteristics of Reacted Goethite

Notably, reacted goethite samples were treated with 30% H_2_O_2_ at 60 °C for 24 h to remove adsorbed organic matter prior to FTIR characterization. Although this oxidation procedure may modify surface hydroxyl groups, surface hydroxylation state, and Fe redox state, all samples (including the blank CK) received identical H_2_O_2_ treatment. Thus, the relative differences in peak intensities between treatments reflect reaction-induced changes rather than cleaning artifacts. The 3104 cm^−1^ band, assigned to structural ≡Fe–OH stretching, exhibited significantly higher intensity in the CK group than in all experimental groups. This arose from extensive ligand exchange between organic precursors and surface Fe^3+^ ions, which consumed free ≡Fe–OH groups and converted them into bound moieties within mineral–organic complexes [58,59]. This ligand exchange process not only concentrated organic precursors at the mineral–water interface but also lowered the activation energy for subsequent electron transfer and oxidative polymerization reactions, constituting the initial and rate-limiting step of goethite catalysis [58,59]. Notably, the Gly series exhibited a distinct unimodal intensity trend peaking at 0.12 mol/L. At low to moderate Gly concentrations, Gly molecules preferentially form monodentate complexes with high-energy ≡Fe–OH sites, inducing subtle changes in the local lattice environment that exposed additional surface hydroxyl groups and increased overall catalytic activity. However, when Gly concentration exceeded 0.12 mol/L, excess molecules competed for limited surface sites and formed multidentate complexes that consumed multiple ≡Fe–OH groups per adsorbed molecule, resulting in a net decrease in free active sites and reduced reaction efficiency. This was consistent with the kinetic analysis showing the shortest humification active period at this optimal concentration [60].

The 1785 cm^−1^ peak, assigned to Fe–O–H bending vibration of goethite surface hydroxyl groups, was negligible in the CK group but prominent in all treatments, confirming that precursor-induced interfacial reactions significantly altered the local chemical environment of surface Fe–O–H moieties on goethite. Based on previous studies, a plausible redox pathway involves electron transfer from catechol to surface Fe(III), producing semiquinone/quinone intermediates and transient Fe(II). Subsequent Fe(II)/Fe(III) cycling and possible electron hopping within iron oxides may facilitate electron transfer to molecular oxygen and generate H_2_O_2_ or other reactive oxygen species, supporting POD-like or Fenton-like oxidation [49,50]. However, these processes were not directly measured in this study, and Fe(II), H_2_O_2_, reactive oxygen species and dissolved oxygen consumption were not quantified. Accordingly, the above redox pathways remain mechanistic hypotheses that require further experimental verification. Additionally, the H_2_O_2_ cleaning procedure applied to reacted goethite may oxidize residual Fe(II), alter surface hydroxylation and transform mineral-bound organic matter, which may modify the original surface features induced by precursor reactions. Therefore, the FTIR results cannot unambiguously demonstrate substrate-induced goethite surface reconstruction, and only reflect changes in surface functional group environments after oxidative cleaning. Surface Fe^3+^ ions were proposed to act as strong electron acceptors and drive three parallel oxidation pathways: Glu ring-opening and sequential dehydration to aldehydes/ketones, Cat oxidation to semiquinone radicals and quinones, and Gly oxidative deamination to glyoxylic acid [14]. These reactive carbonyl intermediates acted as critical molecular hubs connecting the two core humification pathways. Carbonyl groups derived from Glu and glyoxylic acid underwent nucleophilic addition with Gly amino groups to form Schiff bases and Amadori products in the Maillard reaction, while quinones served as electron acceptors and cross-linking agents that covalently bonded with Maillard intermediates to form high-molecular-weight HLA with complex aromatic networks [31,61]. The 1652 cm^−1^ band, attributed predominantly to adsorbed water H–O–H bending vibration on goethite surfaces. Its intensity variation across treatments reflected differences in the abundance of surface-adsorbed water, which was tightly linked to the surface hydroxylation degree and organic ligand coordination state of goethite. Minor overlapping contributions from residual aromatic C=C stretching and amide C=O stretching of trace mineral-bound organic matter cannot be fully excluded, but adsorbed water bending is the dominant origin of this band in H_2_O_2_-cleaned goethite samples [22,23,24,25,26]. Finally, enhanced 890 cm^−1^ and 796 cm^−1^ intensities in all treatments demonstrated substrate-induced modulation of goethite surface functional groups. Organic ligand coordination distorted the crystal lattice, exposed additional active sites, and rigidified the mineral surface, creating a self-amplifying catalytic loop that accelerated humification. Direct evidence for lattice-level reconstruction (e.g., from XRD, XPS, or high-resolution electron microscopy) is still lacking, and this mechanistic hypothesis requires further verification in future studies.

### 3.5. PLS-SEM and Mechanistic Insights

The dominant role of precursor concentration in driving reaction kinetics (standardized path coefficient = 0.900) was consistent with fundamental chemical kinetics principles: higher substrate concentrations increased the frequency of intermolecular collisions between amino groups, carbonyl groups, and phenolic moieties. This finding was in line with prior qualitative reports that precursor availability limited abiotic humification rate [15,16]. The 322.66% increase in DOC accumulation in the Glu series across the concentration gradient demonstrated that C input intensity was the primary determinant of total humification extent under mineral-catalyzed conditions. This effect operated through accelerating both the initial condensation steps of the Maillard reaction and the subsequent oxidative polymerization of polyphenolic intermediates, which were the two core reactions driving abiotic HS formation.

The exceptionally strong positive effect of reaction kinetics on DOC accumulation efficiency highlighted the primacy of reaction completeness in determining DOC accumulation. This pathway represented the quantity dimension of product formation, where faster and more extensive conversion of soluble precursors into insoluble HLS directly increased the total C pool retained in the system. Importantly, this effect was sufficiently strong to override the negative impacts of reduced humification maturity observed at high substrate concentrations, which explained why high-input systems consistently achieved higher total DOC accumulation despite lower product quality. Complementing the quantity pathway, the strong positive effect of product structure on DOC accumulation efficiency underscored the critical importance of molecular properties for long-term carbon stability. It should be emphasized that long-term stability, biodegradation resistance, and in-situ soil carbon sequestration potential were not measured in this study. HLS with higher aromaticity and greater N enrichment are hypothesized to exhibit enhanced chemical recalcitrance due to the presence of stable aromatic ring structures and heterocyclic N moieties, which were less susceptible to enzymatic degradation by soil microorganisms [5,62]. This finding confirmed the central tenet of soil C stabilization theory that molecular structure was a primary controller of organic matter persistence, and demonstrated that abiotic humification could directly modulate C sequestration potential by altering the molecular architecture of humic products.

The unexpected positive effect of precursor concentration on mineral interface activity challenged the conventional site competition paradigm, which posited that organic molecules passively occupied and blocked mineral active sites. Instead, our results supported a dynamic surface reconstruction mechanism, where low to moderate concentrations of organic ligands induced localized dissolution and recrystallization of goethite surfaces. This process exposed additional edge and defect sites that possessed higher catalytic activity for oxidation and polymerization reactions. This observation revised our understanding of mineral–organic interactions during humification, revealing that organic substrates could actively modify mineral catalytic properties rather than merely being passively adsorbed.

The negative relationship between reaction kinetics and product composition revealed a fundamental thermodynamic and kinetic constraint in abiotic humification systems. This rate–quality trade-off originated from the finite number of catalytically active sites on the goethite surface. At low substrate concentrations, all precursor molecules could access these sites and undergo sequential, catalyzed polymerization reactions that favored the formation of high-molecular-weight HLA. At high substrate concentrations, however, excess precursors underwent rapid, uncatalyzed polymerization in the aqueous phase, producing predominantly low-molecular-weight FLA with lower humification maturity.

The relatively weak effect of mineral interface activity on reaction kinetics defined the operational boundary of goethite catalysis in humification. Under the substrate-sufficient conditions employed in this study, reaction rates were primarily limited by the availability of reactive precursors rather than the concentration of mineral catalytic sites. This context-dependent catalytic role implied that goethite exerted a much stronger influence on humification rates in oligotrophic environments where substrate concentrations were low, and mineral surfaces served as the primary sites for concentrating and activating dilute organic molecules.

Finally, the absence of a direct effect of reaction kinetics on product structure provided compelling evidence that HLS quantity maturity (assessed by the C_HLA_/C_FLA_ ratio) and structural maturity (assessed by aromaticity and elemental composition) were decoupled processes. This finding had profound implications for HLS characterization, as it demonstrated that traditional indices of humification maturity based on component fractions did not reliably predict the molecular properties that determined long-term C sequestration potential. Instead, molecular-level characterization was essential for accurately evaluating the stability of HLS formed under different environmental conditions.

To clarify the scope of this study, we explicitly distinguish four hierarchical processes involved in mineral-mediated organic C transformation:

(1) Rapid adsorption and ligand exchange: Precursors bind to goethite surface ≡Fe–OH sites via hydrogen bonding, ligand exchange and electrostatic interaction, which is the initial and core regulatory step of interfacial reactions; (2) Mineral-mediated electron transfer and oxidative transformation: Surface Fe(III) acts as an electron acceptor to drive precursor oxidation, and potential Fe redox cycling and reactive oxygen species generation further promote oxidative polymerization; (3) Condensation polymerization and operational HLA/FLA formation: Intermediates undergo cross-linking and polymerization to form operationally defined HLA and FLA fractions, which can be separated by acid-base extraction; (4) Long-term humification and persistent SOC stabilization: Humic substances undergo long-term structural evolution, form stable mineral–organic associations, and exhibit resistance to microbial degradation and desorption, achieving long-term C sequestration.

This 360-h incubation study covers the first three processes and characterizes the short-term mineral–organic interfacial transformation features. The conclusions of this study are limited to short-term goethite-mediated formation of operationally defined humic-like products, and cannot directly confirm long-term soil C sequestration effects.

Methodological limitations should also be noted. The 0.2 mol/L phosphate buffer used in this study is not an inert background electrolyte. Phosphate can occupy surface ≡Fe–OH groups, alter surface charge, compete with organic ligands for adsorption sites, and modify interfacial electron transfer behavior. The reactions observed in this study occur on a phosphate-conditioned goethite surface, and caution should be exercised when extrapolating to natural soil environments. We acknowledge that phosphate uptake by goethite was not quantified in this work, and that no low-phosphate, phosphate-free, or non-coordinating-buffer control was included. Therefore, we cannot fully disentangle the independent contributions of phosphate and precursors to goethite interfacial reactivity. Nevertheless, because the phosphate background was completely uniform across all Gly gradient, Glu gradient, and blank CK treatments, the observed concentration-dependent effects of glycine and glucose on reaction kinetics, product composition, and structural properties reflect relative differences measured on the same phosphate-conditioned goethite surface. The influence of phosphate on the mineral surface is equal across all treatments and does not introduce systematic bias between groups, so all between-treatment comparisons remain valid and internally consistent. The conclusions of this study apply specifically to goethite-catalyzed abiotic humification under phosphate-buffered conditions.

This study did not establish a full C mass balance, including CO_2_ evolution, mineral-associated organic C and insoluble precipitate C, so net C retention could not be quantitatively determined. Direct evidence for Fe redox cycling, reactive oxygen species generation and in-situ surface reconstruction was lacking. Furthermore, the current study does not include a substrate-only control without goethite, which precludes the quantitative disentanglement of spontaneous abiotic humification from mineral-catalyzed humification pathways.

## 4. Materials and Methods

### 4.1. Materials

Goethite was synthesized following the classic method described by Atkinson et al. Briefly, 50 g of Fe(NO_3_)_3_·9H_2_O was dissolved in 825 mL of deionized water in a wide-mouth polyethylene bottle. A 2.5 mol/L NaOH solution was then added dropwise under constant magnetic stirring until the suspension pH reached 11.9. The resulting suspension was aged at 60 °C for 48 h in a water bath. The precipitate was harvested via centrifugation and washed repeatedly with deionized water until the electrical conductivity of the supernatant stabilized below 10 μS/cm. The purified goethite was dried at 60 °C, ground to a fine powder, and passed through a 100-mesh sieve before use.

Analytical-grade D-(+)-glucose (Glu) and glycine (Gly, achiral with no L/D isomerism) were obtained from Sinopharm Chemical Reagent Co., Ltd. (Shanghai, China). A 0.2 mol/L phosphate buffer solution (pH 7.0) was prepared by mixing appropriate volumes of NaH_2_PO_4_ and Na_2_HPO_4_ stock solutions. This buffer concentration was selected to maintain a stable pH of 7.0 throughout the 360-h incubation period, as abiotic humification pathways are highly pH-dependent, and a pH shift of 1–2 units would completely obscure the effects of precursor concentration and mineral catalysis. The use of 0.2 mol/L phosphate buffer is a widely accepted standard practice in abiotic humification research to eliminate pH variation as a confounding variable [63,64,65,66]. It should be noted that phosphate ions are not an inert background electrolyte: they can adsorb onto goethite surfaces via ligand exchange, alter surface charge and active site density, and compete with organic precursors for adsorption sites, thereby modulating interfacial electron transfer behavior [64]. However, this surface modification is not an experimental artifact; in natural soils and mineral–water interfaces, iron oxides are ubiquitously coated with phosphate, organic matter, and other anions, and local phosphate concentrations in soil microsites and mineral boundary layers can reach millimolar levels. Accordingly, a phosphate-conditioned goethite surface is more environmentally relevant than an idealized “phosphate-free clean surface”. Critically, all treatments (Gly gradient, Glu gradient, and blank CK) received an identical concentration of 0.2 mol/L phosphate buffer at the same pH, so the phosphate background was completely uniform across all experimental groups. Thimerosal (C_9_H_9_HgNaO_2_S) was added at a final concentration of 0.02% (*w*/*v*) to inhibit microbial activity throughout the entire incubation period.

### 4.2. Experimental Method

All incubation experiments were performed using a sterile liquid shake-flask system. Each 500 mL Erlenmeyer flask contained 250 mL of phosphate buffer and 2 g of synthetic goethite. The experimental design comprised two independent series: Series 1 (Gly gradient series) maintained fixed concentrations of Cat and Glu while varying Gly concentration; Series 2 (Glu gradient series) maintained fixed concentrations of Cat and Gly while varying Glu concentration. A blank control (CK) containing only phosphate buffer and goethite was also included to quantify background C release from the mineral phase. Notably, a substrate-only control without goethite was not included in the formal experimental design. Prior preliminary tests demonstrated that the system containing only the three Maillard precursors (Cat, Gly and Glu) in the absence of goethite remained a clear aqueous solution throughout the 360-h incubation period, with no dark-brown residue formed. Correspondingly, neither HLA nor FLA fractions could be isolated using the standard acid precipitation-extraction protocol employed in this study. Given that the substrate-only control produced no measurable products consistent with the operational definitions of the humification indicators used in this work, its contribution to the quantitative analysis of concentration-dependent regulatory patterns was deemed very limited. For the Gly gradient series, Gly was added at final concentrations of 0, 0.03, 0.06, 0.12, and 0.24 mol/L, while Glu and Cat were maintained at fixed concentrations of 0.06 mol/L each; these conditions were designated as Gly0, Gly0.03, Gly0.06, Gly0.12, and Gly0.24, respectively. Similarly, for the Glu gradient series, Glu was applied at the same concentration gradient (0, 0.03, 0.06, 0.12, and 0.24 mol/L), and fixed Gly and Cat concentrations (0.06 mol/L each), with Gly and Cat held constant at 0.06 mol/L each; corresponding designations were Glu0, Glu0.03, Glu0.06, Glu0.12, and Glu0.24. All treatments were established in triplicate.

All flasks were autoclaved at 121 °C for 20 min. After cooling to room temperature, the flasks were incubated in the dark at 28 °C with rotary shaking at 150 rpm. Aliquots of suspension were collected aseptically at 0, 3, 6, 18, 28, 48, 76, 124, 172, 240, and 360 h. At each sampling time point, 2 mL of suspension was withdrawn aseptically and centrifuged at 16,000× *g* for 5 min. A 1 mL aliquot of the supernatant was diluted to 25 mL with sterile phosphate buffer, and absorbance values were measured at 465 nm (E_4_) and 665 nm (E_6_). The remaining supernatant was stored at 4 °C prior to DOC analysis.

HLS fractionation was performed at 0, 18, 48, 76, 124, 172, 240, and 360 h using 10 mL of suspension per sample. After centrifugation at 16,000× *g* for 10 min, the supernatant was separated from the mineral pellet. The pellet was acidified to pH 1.0 with 1.0 mol/L HCl, equilibrated for 24 h at room temperature, and centrifuged a second time. The resulting supernatant, defined as the fulvic-like acid (FLA) fraction, was neutralized and diluted to a constant volume. The residual pellet after acidification was designated as the humic-like acid (HLA) fraction. The HLA fraction was redissolved in 0.1 mol/L NaOH at 60 °C, neutralized, and adjusted to a fixed volume. The organic C concentrations of both HLA and FLA solutions were quantified using a Model vario TOC analyzer (Elementar Analysensysteme GmbH, Langenselbold, Hesse, Germany).

For solid HLA sample preparation, the reaction mixture after 360 h of incubation was centrifuged. HLA adsorbed on the pellet was extracted with 0.1 mol/L NaOH, and residual goethite particles were removed via centrifugation. The supernatant was subsequently treated with a mixed solution of 0.1 mol/L HCl and 0.3 mol/L HF for 24 h to remove residual inorganic impurities. The purified HLA precipitate was freeze-dried, ground to a fine powder, and passed through a 0.01 mm sieve for subsequent elemental analysis and FTIR characterization. After 360 h of incubation, the mineral precipitates remaining after HLA extraction were rinsed with 30% H_2_O_2_ at 60 °C for 24 h to remove all adsorbed organic matter, allowing characterization of the intrinsic functional groups of the reacted goethite. It should be noted that this H_2_O_2_ oxidation treatment may alter surface hydroxyl groups, surface hydroxylation state, and Fe redox state. The magnitude of these effects was not experimentally quantified. All samples (including the blank CK control) underwent identical cleaning procedures, so that relative between-treatment differences in FTIR signals are attributable to reaction effects rather than cleaning artifacts.

### 4.3. Analytical Methods

Absorbance values at E_4_ and E_6_ for both the supernatant and diluted HLA solutions were measured using a UV–Vis spectrophotometer (TU-1900, Beijing Purkinje General Instrument Co., Ltd., Beijing, China), and the E_4_/E_6_ ratio was calculated accordingly. The elemental composition (mass fractions of C, H, O, and N) of the solid HLA samples was determined using an elemental analyzer (PerkinElmer PE 2400 II CHNS/O, PerkinElmer, Waltham, MA, USA). All measurements were performed in triplicate, and results are reported as mean values.

FTIR spectra of the dark-brown HLA residues and reacted goethite samples were recorded using an FTIR spectrophotometer (Model FTIR-850, Tianjin Gangdong Technology Development Co., Ltd., Tianjin, China). Spectra were collected across the wavenumber range of 4000–400 cm^−1^ using the KBr pellet method (1 mg sample mixed with 200 mg dried spectroscopic-grade KBr), with a spectral resolution of 4 cm^−1^ and 32 scans per sample. Spectral processing and analysis were performed using the FTIR850 software package (Version 2.0, Tianjin Gangdong Technology Development Co., Ltd., Tianjin, China). All graphical visualizations were generated using Origin 8.0 (OriginLab, Northampton, MA, USA).

Data processing and spectral analysis were performed using Microsoft Excel 2003 (Microsoft Corporation, Redmond, WA, USA) and Origin 8.0. Statistical analyses were carried out using SPSS 18.0 (IBM, Armonk, NY, USA). Prior to analysis of variance (ANOVA), data normality was verified via the Shapiro–Wilk test, and homogeneity of variance was confirmed using Levene’s test. A *p*-value < 0.05 was considered statistically significant. Significant differences among treatments were assessed via one-way ANOVA, followed by Fisher’s least significant difference (LSD) post-hoc test.

PLS-SEM was conducted using the plspm package (Version 0.4.9) in R 4.5.1 (R Foundation for Statistical Computing, Vienna, Austria) to elucidate the rate–quality trade-off in precursor-driven, goethite-mediated humification. The model goodness-of-fit (GoF) was calculated, and the significance of path coefficients was tested via 1000 two-tailed bootstrap resampling iterations.

## 5. Conclusions

This study systematically elucidated the divergent regulatory mechanisms of Gly and Glu concentration gradients in short-term goethite-mediated coupled polyphenol–Maillard interfacial transformation. Gly modulated the aromatic condensation degree of HLS primarily by supplying amino groups to construct N-containing aromatic heterocycles, whereas Glu served as the predominant aliphatic C skeleton donor that governed the stable DOC generation potential of the reaction system. A concentration of 0.12 mol/L was identified as the optimal matching concentration for both precursors, balancing mineral interfacial adsorption capacity and humification transformation efficiency. Increasing Gly content continuously enriched N in HLA and decreased its C/N ratio. In contrast, elevated Glu levels promoted the accumulation of aliphatic C chains, thereby increasing the C/N ratio of HLA. Excessive concentrations of both precursors induced competitive adsorption on ≡Fe–OH active sites of goethite surfaces, which inhibited the transformation of labile FLA into stable HLA fractions and reduced overall humification maturity. FTIR spectroscopic characterization verified that Gly consumed free hydroxyl groups through amide and heterocycle formation, while Glu facilitated the accumulation of aliphatic hydroxyl intermediates. Both precursors modulated the surface functional group composition and reactivity of goethite, and activated Fe-bearing surface functional groups. However, FTIR data alone cannot provide direct evidence for crystal lattice reconstruction or dissolution-recrystallization processes; these mechanistic hypotheses require validation via complementary structural characterization techniques (e.g., XRD, XPS, electron microscopy) in future work. The revised PLS-SEM analysis with independent indicators confirmed that precursor concentration acted as the core driver governing humification reaction kinetics. Overall, this study clarifies the differential interfacial catalytic pathways of amino acid and sugar precursors during short-term mineral–organic interaction, advances the mechanistic understanding of iron-mediated abiotic transformation of organic precursors, and provides quantitative benchmarks for optimizing artificial HS production processes. Long-term stability assays, biodegradation tests, and full C mass balance are required to verify the long-term SOC sequestration potential of the products.

## Figures and Tables

**Table 1:** Gaussian model fitting parameters for the temporal dynamics of the E_4_/E_6_ ratio under different Gly and Glu concentrations.

Treatment	*y*_0_	*x_c_*	*A*	*w*	Coefficient (R^2^)
Gly0	1.31039	16.61733	26.31945	9.06221	0.89753
Gly0.03	1.86233	16.42011	23.7882	10.72844	0.99983
Gly0.06	2.27069	15.15471	37.13489	6.78143	0.9998
Gly0.12	2.45297	20.70516	73.77369	5.84364	0.88752
Gly0.24	2.73717	21.09759	41.6637	5.96528	0.99991
Glu0	1.75788	21.43066	43.38573	6.71857	0.9981
Glu0.03	1.87054	18.00000	9.87734	2.77348	0.91667
Glu0.06	2.07714	17.81478	15.03324	2.21507	0.94433
Glu0.12	1.94817	18.0000	7.12549	1.86673	0.99988
Glu0.24	2.11625	20.62358	40.48473	5.62143	0.63758
Control (CK)	34.53256	219.62115	−90,682.75785	2500.09094	0.58286

Standard errors of all Gaussian-fitted parameters are provided in Table S1 in the Supporting Information. The relative standard errors of *y*_0_, *x_c_*, *A*, and *w* were all below 6% for precursor-amended treatments, indicating high reliability of the fitted parameters for cross-treatment comparisons.

**Table 2:** First-order asymptotic model fitting parameters for supernatant DOC dynamics under different Gly and Glu concentrations.

Treatment	*a*	*b*	*c*	Coefficient (R^2^)
Gly0	7.7517	−7.58241	0.61397	0.9992
Gly0.03	8.60515	−7.69975	0.65580	0.99928
Gly0.06	9.05049	−9.91482	0.55874	0.9998
Gly0.12	10.663	−12.4166	0.55319	0.99869
Gly0.24	13.79955	−15.99878	0.69582	0.99895
Glu0	5.34777	−6.16129	0.60770	0.99719
Glu0.03	7.1824	−5.48455	0.61375	0.99768
Glu0.06	9.96959	−9.57394	0.54364	0.99904
Glu0.12	14.29915	−10.6779	0.63003	0.99942
Glu0.24	22.60267	−24.22545	0.52738	0.99939
CK	0.74225	0.49174	0.91793	0.99575

Standard errors of all first-order asymptotic model parameters are provided in Table S2. The relative standard errors of a and b were below 4% for all treatments, supporting the validity of the percentage change calculations.

**Table 3:** Atomic molar ratios of HLA extracted from dark-brown residues produced via goethite-mediated Maillard reactions under varying Gly and Glu concentrations.

Treatments	H/C Ratio	C/N Ratio	O/C Ratio
Gly0	0.95 ± 0.06 e	29.78 ± 0.36 a	1.03 ± 0.01 d
Gly0.03	1.36 ± 0.03 d	20.26 ± 0.10 b	1.46 ± 0.01 b
Gly0.06	1.57 ± 0.05 c	17.23 ± 0.11 c	1.60 ± 0.03 c
Gly0.12	1.72 ± 0.08 b	8.64 ± 0.12 d	2.40 ± 0.02 a
Gly0.24	1.88 ± 0.06 a	7.91 ± 0.11 e	2.45 ± 0.02 a
Glu0	1.29 ± 0.05 c	14.77 ± 0.10 e	1.32 ± 0.04 c
Glu0.03	1.36 ± 0.04 b	15.31 ± 0.06 d	1.39 ± 0.01 b
Glu0.06	1.40 ± 0.05 b	15.59 ± 0.18 c	1.43 ± 0.01 b
Glu0.12	1.41 ± 0.05 b	16.09 ± 0.10 b	1.52 ± 0.02 d
Glu0.24	1.58 ± 0.07 a	17.20 ± 0.13 a	1.72 ± 0.03 a

Note: Different lowercase letters in the same column indicate significant differences among treatments at *p* < 0.05 (LSD test).

**Table 4:** Relative FTIR peak intensities (% of total integrated area) of dark-brown residues generated via the Maillard reaction under different Gly and Glu concentrations.

Treatment	3426 cm^−1^	1630 cm^−1^	890 cm^−1^	790 cm^−1^
	–OH	C=C & H–O–H	Surface Fe–OH	Fe–O–Fe
Gly0	76.30%	17.50%	0.90%	1.40%
Gly0.03	75.00%	19.40%	2.60%	2.60%
Gly0.06	72.90%	20.00%	1.80%	1.80%
Gly0.12	70.80%	21.30%	3.70%	3.00%
Gly0.24	67.90%	24.10%	2.70%	2.70%
Glu0	69.30%	17.10%	3.00%	2.80%
Glu0.03	70.50%	19.90%	2.90%	2.90%
Glu0.06	72.60%	21.90%	1.90%	2.00%
Glu0.12	77.40%	19.20%	3.50%	3.30%
Glu0.24	82.00%	17.10%	3.00%	2.80%
CK	65.60%	16.30%	0.00%	0.50%

**Table 5:** Relative FTIR peak intensities (% of total integrated area) of reacted goethite from the Maillard reaction system under different Gly and Glu concentrations.

Treatment	3104 cm^−1^	1785 cm^−1^	1652 cm^−1^	890 cm^−1^	796 cm^−1^
	≡Fe–OH	Fe–O–H Bending Vibration	Adsorbed Water H–O–H Bending	Surface Fe–OH	Fe–O–Fe
Gly0	28.8 a	2.6 c	7.9 c	33.1 f	20.7 d
Gly0.03	30.3 e	2.2 b	6.0 a	30.5 b	19.5 b
Gly0.06	32.3 b	2.6 c	7.0 b	32.2 d	20.9 cd
Gly0.12	33.5 c	2.5 c	6.5 b	32.2 c	20.8 c
Gly0.24	32.9 d	2.4 b	6.3 a	32.9 e	21.3 cd
Glu0	33.6 c	2.3 b	7.6 c	31.6 c	20.0 b
Glu0.03	32.3 a	2.6 c	7.1 b	32.8 e	20.9 c
Glu0.06	36.4 d	2.4 b	6.3 a	30.8 b	20.2 b
Glu0.12	32.0 a	2.5 c	7.6 c	32.4 c	20.6 c
Glu0.24	32.1 b	2.3 b	6.8 b	32.2 d	21.0 d
CK	41.2 f	0.8 a	5.7 b	18.1 a	14.9 a

Note: Different lowercase letters in the same column indicate significant differences among treatments at *p* < 0.05 (LSD test).

**Table 6:** Latent variables and corresponding observed indicators of the PLS-SEM model.

Model Variables	Observed Indicators
Precursor concentration	Precursor concentration (mol/L)
Mineral interface	Relative peak area of 3104 cm^−1^ and 890 cm^−1^ from reacted goethite FTIR spectra
Reaction kinetics	Supernatant E_4_/E_6_ Gaussian model parameters *x_c_* and *w*; DOC asymptotic growth model rate constant *c*
Product composition	C_HLA_ at 360 h, C_HLA_/C_FLA_ ratio
Product structure	HLA H/C ratio, C/N ratio, and relative peak area of 1630 cm^−1^ from dark-brown residues FTIR spectra
DOC accumulation	Measured DOC concentration in supernatant at 360 h

## Data Availability

Data will be made available on request.

## Supplementary Materials

The following supporting information can be downloaded at: https://www.mdpi.com/article/10.3390/molecules31183302/s1, Table S1. Gaussian model parameters for E_4_/E_6_ ratio temporal dynamics. Table S2. First-order asymptotic model parameters for supernatant DOC temporal dynamics.
