# Supplementary material for: Glycine and Glucose Exert Divergent Concentration-Dependent Interfacial Regulatory Effects on Goethite-Catalyzed Polyphenol–Maillard Abiotic Humification
Source: Molecules. 2026 Sep 17;31(18):3302. doi: 10.3390/molecules31183302 (PMC13609666; doi:10.3390/molecules31183302)
Supplement: Supplementary file 1 [file molecules-31-03302-s001.zip › molecules-4494828-supplementary.pdf]

Supporting Information

Table S1. Gaussian model parameters for E_4/E_6 ratio temporal dynamics

Treatment	Y_0 (mean ± SE)	x_c (mean ± SE, h)	A (mean ± SE)	w (mean ± SE, h)
Gly0	1.31039 ± 0.042	16.61733 ± 0.72	26.31945 ± 1.25	9.06221 ± 0.41
Gly0.03	1.86233 ± 0.051	16.42011 ± 0.65	23.7882 ± 1.01	10.72844 ± 0.46
Gly0.06	2.27069 ± 0.058	15.15471 ± 0.57	37.13489 ± 1.63	6.78143 ± 0.29
Gly0.12	2.45297 ± 0.085	20.70516 ± 0.98	73.77369 ± 3.52	5.84364 ± 0.27
Gly0.24	2.73717 ± 0.073	21.09759 ± 0.92	41.6637 ± 1.95	5.96528 ± 0.28
Glu0	1.75788 ± 0.053	21.43066 ± 0.95	43.38573 ± 2.03	6.71857 ± 0.32
Glu0.03	1.87054 ± 0.069	18.0000 ± 0.76	9.87734 ± 0.53	2.77348 ± 0.15
Glu0.06	2.07714 ± 0.072	17.81478 ± 0.74	15.03324 ± 0.78	2.21507 ± 0.12
Glu0.12	1.94817 ± 0.056	18.0000 ± 0.71	7.12549 ± 0.38	1.86673 ± 0.10
Glu0.24	2.11625 ± 0.081	20.62358 ± 0.97	40.48473 ± 2.11	5.62143 ± 0.30
Control (CK)	34.53256 ± 4.21	219.62115 ± 28.7	−90682.76 ± 12540.18	2500.09 ± 342

Note: The relatively large standard errors for the CK group are consistent with the poor fitting performance of the Gaussian model for the blank system, as noted in the main text.

Table S2. First-order asymptotic model parameters for supernatant DOC temporal dynamics

**Treatment**	***a* (mean ± SE, mg/L)**	***b* (mean ± SE, mg/L)**	***c* (mean ± SE)**
Gly0	7.7517 ± 0.19	−7.5824 ± 0.19	0.6140 ± 0.021
Gly0.03	8.6052 ± 0.22	−7.6998 ± 0.20	0.6558 ± 0.022
Gly0.06	9.0505 ± 0.24	−9.9148 ± 0.26	0.5587 ± 0.018
Gly0.12	10.6630 ± 0.31	−12.4166 ± 0.36	0.5532 ± 0.019
Gly0.24	13.7996 ± 0.38	−15.9988 ± 0.44	0.6958 ± 0.024
Glu0	5.3478 ± 0.15	−6.1613 ± 0.17	0.6077 ± 0.021
Glu0.03	7.1824 ± 0.20	−5.4846 ± 0.15	0.6138 ± 0.020
Glu0.06	9.9696 ± 0.27	−9.5739 ± 0.26	0.5436 ± 0.018
Glu0.12	14.2992 ± 0.39	−10.6779 ± 0.29	0.6300 ± 0.021
Glu0.24	22.6027 ± 0.62	−24.2255 ± 0.66	0.5274 ± 0.017
Control (CK)	0.7423 ± 0.031	0.4917 ± 0.022	0.9179 ± 0.035
